# Alleviation of *Limosilactobacillus reuteri* in polycystic ovary syndrome protects against circadian dysrhythmia-induced dyslipidemia via capric acid and GALR1 signaling

**DOI:** 10.1038/s41522-023-00415-2

**Published:** 2023-07-08

**Authors:** Shang Li, Junyu Zhai, Weiwei Chu, Xueying Geng, Dongshuang Wang, Luwei Jiao, Gang Lu, Wai-Yee Chan, Kang Sun, Yun Sun, Zi-Jiang Chen, Yanzhi Du

**Affiliations:** 1 Center for Reproductive Medicine, Ren Ji Hospital, School of Medicine, Shanghai Jiao Tong University, Shanghai, 200135 China; 2 Shanghai Key Laboratory for Assisted Reproduction and Reproductive Genetics, Shanghai, 200135 China; 3 The Chinese University of Hong Kong-Shandong University Joint Laboratory on Reproductive Genetics, School of Biomedical Sciences, The Chinese University of Hong Kong, Hong Kong SAR, China; 4 Center for Reproductive Medicine, Shandong University, National Research Center for Assisted Reproductive Technology and Reproductive Genetics, Key Laboratory of Reproductive Endocrinology of Ministry of Education, Shandong Provincial Key Laboratory of Reproductive Medicine, Jinan, Shandong 250012 China; 5 NMU-SD Suzhou Collaborative Innovation Center for Reproductive Medicine, Suzhou, Jiangsu, China

**Keywords:** Microbiota, Microbiome

## Abstract

Knowledge gaps that limit the development of therapies for polycystic ovary syndrome (PCOS) concern various environmental factors that impact clinical characteristics. Circadian dysrhythmia contributes to glycometabolic and reproductive hallmarks of PCOS. Here, we illustrated the amelioration of *Limosilactobacillus reuteri* (*L. reuteri*) on biorhythm disorder-ignited dyslipidemia of PCOS via a microbiota-metabolite-liver axis. A rat model of long-term (8 weeks) darkness treatment was used to mimic circadian dysrhythmia-induced PCOS. Hepatic transcriptomics certified by in vitro experiments demonstrated that increased hepatic galanin receptor 1 (GALR1) due to darkness exposure functioned as a critical upstream factor in the phosphoinositide 3-kinase (PI3K)/protein kinase B pathway to suppress nuclear receptors subfamily 1, group D, member 1 (NR1D1) and promoted sterol regulatory element binding protein 1 (SREBP1), inducing lipid accumulation in the liver. Further investigations figured out a restructured microbiome-metabolome network following *L. reuteri* administration to protect darkness rats against dyslipidemia. Notably, *L. reuteri* intervention resulted in the decrease of *Clostridium sensu stricto 1* and *Ruminococcaceae UCG-010* as well as gut microbiota-derived metabolite capric acid, which could further inhibit GALR1-NR1D1-SREBP1 pathway in the liver. In addition, GALR antagonist M40 reproduced similar ameliorative effects as *L. reuteri* to protect against dyslipidemia. While exogenous treatment of capric acid restrained the protective effects of *L. reuteri* in circadian disruption-induced PCOS through inhibiting GALR1-dependent hepatic lipid metabolism. These findings purport that *L. reuteri* could serve for circadian disruption-associated dyslipidemia. Manipulation of *L. reuteri*–capric acid–GALR1 axis paves way for clinical therapeutic strategies to prevent biorhythm disorder-ignited dyslipidemia in PCOS women.

## Introduction

Polycystic ovary syndrome (PCOS), a prevalent disorder of the reproductive and endocrine systems presents with a triad of hyperandrogenism, chronic anovulation, and polycystic ovaries. Other than infertility associated with anovulation, women with PCOS are more susceptible to metabolic syndrome that includes obesity, insulin resistance, and hyperlipidemia thus predisposing them type 2 diabetes mellitus, non-alcoholic fatty liver disease (NAFLD), cardiovascular diseases, etc.^1^. The incidence of dyslipidemia in women with PCOS ranges from 50–70%^2,3^. Adolescent daughters of PCOS women are also likely to have increased triglyceride (TG) level^4^. Eighty-one percent (81%) of women with both PCOS and insulin resistance also have lipid disorders^5^. Hyperlipidemia shares a close link with insulin resistance and hyperandrogenism which are cardinal features of PCOS, thus propagating a vicious cycle^6^. Hence, it is essential to identify preventive and therapeutic strategies against dyslipidemia to address PCOS by investigating the molecular mechanisms driving PCOS.

Hormonal imbalance and metabolic dysregulation orchestrate PCOS, triggered by both environmental factors and genetic predisposition. Circadian rhythm coordinates energy homeostasis and reproductive functions with environmental cues^7,8^. A deterioration of an individual’s sleep/wake pattern marks circadian disruption and relates to increased risk of diseases^9^. An evident correlation has been observed between night shift work and PCOS^10^. Sleep disorders are more likely to exist in PCOS women^11^. A misalignment of the morning circadian rhythm in obese PCOS is associated with suboptimal insulin sensitivity and higher serum free testosterone level^12^. Constant darkness is considered as a circadian metabolic signal^13^. We have recently elucidated that constant darkness treatment induces the glycometabolic and reproductive hallmarks of PCOS in rats^14^. Notably, the characteristic decrease in expressions of circadian clock genes in darkness-treated rats is consistent to that in women with PCOS^14^. Hence, constant darkness exposure rat model might provide unique insights into preventive and therapeutic strategies against circadian dysrhythmia-induced PCOS phenotypes. Yet, scant research focused on potential interventions to PCOS ignited by circadian dysrhythmia exists.

Apart from lifestyle modifications (i.e., dietary restrictions and exercise) recommended as first-line therapy in the clinical management of PCOS^15^, a promising intervention to dyslipidemia is probiotic or synbiotic supplementation based on the theory that dysbiosis of gut microbiota drives the development of PCOS^16,17^. Recent meta-analyses indicate that the administration of a probiotic or synbiotic capsule of mixed ingredients to women with PCOS has a beneficial effect on TG, insulin sensitivity, hyperandrogenism, etc.^18–20^. While the effects of single strain from the mixed probiotics on PCOS as well as applicable PCOS subtypes are not yet clear. *Lactobacillus* is one of the main ingredients of the probiotic capsules used in current clinical trials of PCOS intervention. *Lactobacillus* transplantation could partially relieve the abnormal estrous cycle and ovarian morphology of letrozole-induced PCOS-like rats as well as reducing androgen synthesis^21^. *Limosilactobacillus reuteri* (*L. reuteri*) reportedly plays an imperative role in the maintenance of host lipid metabolism, insulin sensitivity, inflammatory status, immune homeostasis, etc.^22–24^. In a previous study, we have illustrated an improvement in glucose tolerance among rats with constant darkness-induced PCOS after receiving *L. reuteri*^14^. However, the specific mechanisms linking *L. reuteri* to PCOS in relation to circadian rhythms and dyslipidemia remain to be elucidated.

Herein, we investigated the impact of administering *L. reuteri* on dyslipidemia in constant darkness-derived PCOS-like rats and explored the potential mechanisms. We did this using a multiomics analysis of the liver transcriptome, gut microbiome, faecal, and serum metabolomes, with verification in the cell line and rat models. This study will shed light on a novel mechanism for treatment of biorhythm disorder-ignited PCOS and provide a potential therapeutic strategy in clinical settings in the foreseeable future.

## Results

### *L. reuteri* attenuates dyslipidemia in constant darkness-induced PCOS-like rats

**Fig. 1:**
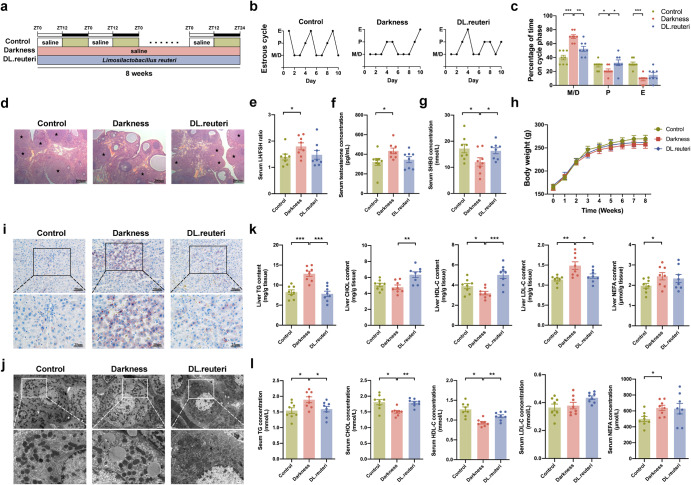
*L. reuteri* supplementation ameliorates dyslipidemia in constant darkness rats. **a** Timeline depicting the treatments of darkness and *L. reuteri* in different groups of the *L. reuteri*-treated rat model. **b** Representative estrous cycles. M, metestrus; D, diestrus; P, proestrus; E, estrus. **c** Quantitative analysis of estrous cycles. **d** Representative hematoxylin and eosin staining of ovaries. Asterisk stands for corpus luteum. Scale bar: 200 μm. **e**–**g** Serum concentrations of LH/FSH ratio (**e**), testosterone (**f**), and SHBG (**g**) detected by ELISA. **h** Body weight changes. **i** Representative Oil Red O staining of livers. Scale bar: 50 μm and 25 μm. **j** Representative images of liver ultrastructure detected by transmission electron microscope. Scale bar: 40 μm and 1 μm. **k** Hepatic contents of TG, CHOL, HDL-C, LDL-C, and NEFA detected by ELISA. **l** Serum concentrations of TG, CHOL, HDL-C, LDL-C, and NEFA detected by ELISA. Statistical analysis was performed with one-way ANOVA followed by Newman–Keuls multiple comparison test. *n* = 8 per group. Data present means ± SEM. *P < 0.05, **P < 0.01, ***P < 0.001.

To understand the effect of *L. reuteri* on circadian dysrhythmia-induced PCOS-like phenotypes, we fed female Sprague–Dawley rats with *L. reuteri* during an 8-week-long constant darkness housing (Fig. 1a). We observed the induction of constant darkness to a PCOS-like rat model with hallmarks including acyclic estrous cycles (Fig. 1b, c), increased numbers of cyst-like follicles and decreased corpora lutea in ovaries (Fig. 1d), elevated serum luteinizing hormone (LH)/ follicle-stimulating hormone (FSH) ratio (Fig. 1e) and testosterone concentration (Fig. 1f), and decreased serum sex hormone-binding globulin (SHBG) level (Fig. 1g), which were consistent with our previous research^14^. Notably, *L. reuteri* administration substantially reversed the disrupted estrous cycles, morphological changes of ovaries, and serum SHBG concentration in darkness rats (Fig. 1b–g). Although there was no difference in body weight among the three groups (Fig. 1h), *L.* *reuteri* prevented the excessive accumulation of lipids in the liver of darkness rats as was shown by Oil Red O staining (Fig. 1i) and transmission electron microscopy (Fig. 1j). Upon analyzing the lipid profiles, darkness rats following *L. reuteri* administration (DL.reuteri rats) presented decreased levels of TG and low-density lipoprotein-cholesterol (LDL-C) as well as increased high-density lipoprotein-cholesterol (HDL-C) level in the liver (Fig. 1k). The high TG level, and low HDL-C and total cholesterol (CHOL) levels in sera of the same rats were also moderated (Fig. 1l). However, *L. reuteri* had no effect on the elevated non-esterified fatty acid (NEFA) level in darkness rats (Fig. 1k, l). Collectively, *L. reuteri* could ameliorate the PCOS-like phenotypes in darkness rats, especially hepatic TG, HDL-C, and LDL-C concentrations as well as serum TG, CHOL, and HDL-C concentrations.

### *L. reuteri* alters hepatic gene expression profile to relieve dyslipidemia in darkness rats

**Fig. 2:**
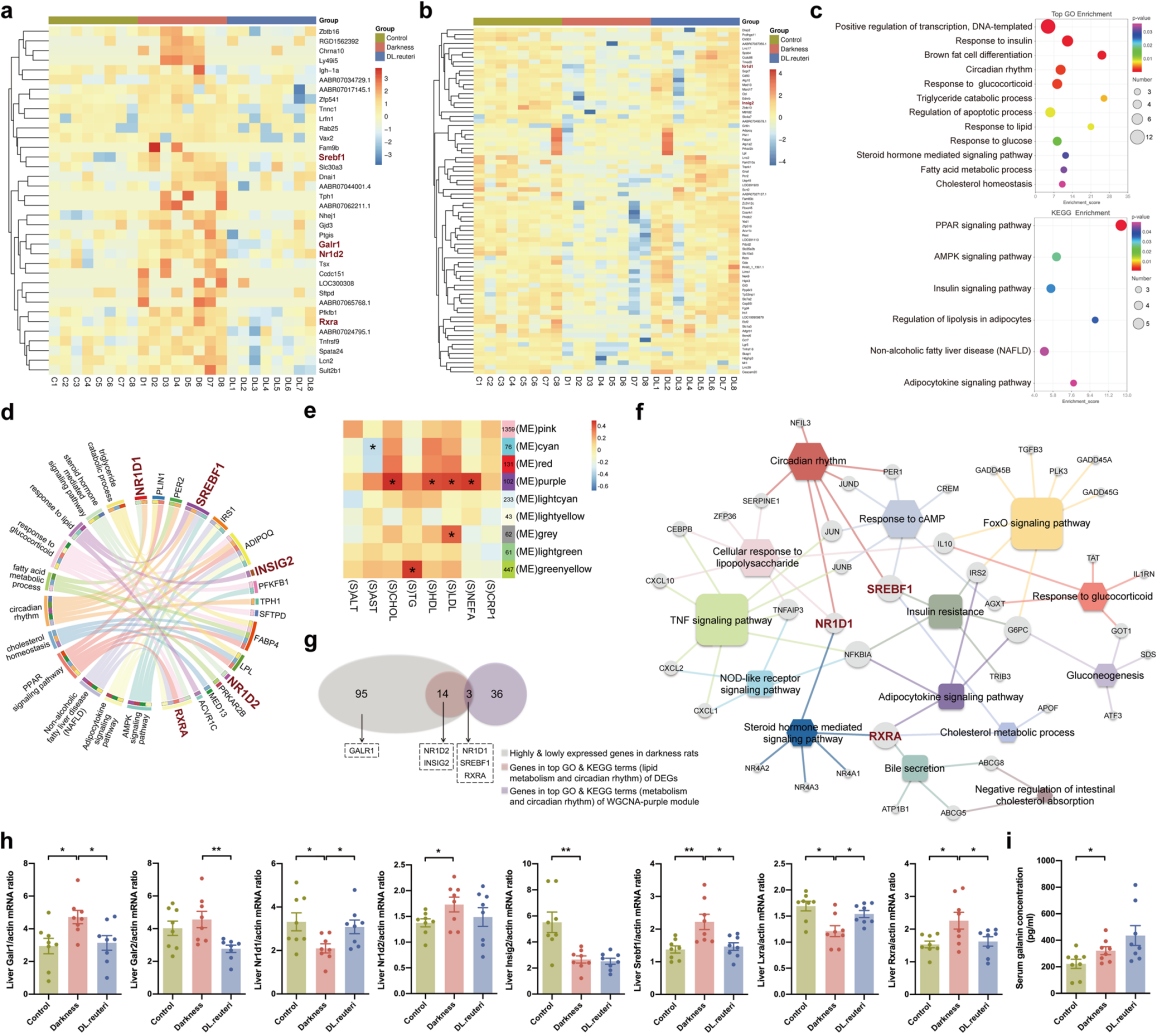
Liver transcriptome analysis of *L. reuteri*-treated darkness rats. Heatmaps displaying 36 highly expressed genes (**a**) and 76 lowly expressed genes (**b**) in the liver of darkness rats compared with control and DL.reuteri rats through DEG analysis using Ballgown software (|fold change | > 0.6 in the log_2_ ratio value, raw *P* < 0.05). **c** Top terms from GO analysis (above) and terms from KEGG analysis (below) of the 112 differential genes in the DEG analysis. The *P* values of GO and KEGG analyses were determined on the DAVID website. **d** Visualization of the top GO and KEGG terms related with lipid metabolism and circadian rhythm in the DEG analysis. **e** Spearman rank correlations between module eigengenes (ME) and clinical biochemical index in the WGCNA analysis (|ρ | > 0.3, *P < 0.05). **f** Visualization of the top GO and KEGG terms related with lipid metabolism and circadian rhythm of purple module genes (102) in the WGCNA analysis. Circle, genes; Square, KEGG terms; Hexagon, GO terms. The bigger the square or hexagon, the more genes involved. **g** Crosstalk among different groups of genes identified the possible target genes taking essential roles in the lipid metabolism of *L. reuteri*-treated darkness rats. **h** mRNA abundances of *Galr1*, *Galr2*, *Nr1d1*, *Nr1d2*, *Insig2*, *Srebf1*, *Lxra*, and *Rxra* in rat liver detected by qPCR. *β-Actin* was used as a loading control for qPCR analyses. **i** Serum galanin concentration detected by ELISA. Statistical analysis (**h,** **i**) was performed with one-way ANOVA followed by Newman–Keuls multiple comparison test. *n* = 8 per group. Data present means ± SEM. *P < 0.05, **P < 0.01.

The observation that *L. reuteri* administration alleviated dyslipidemia and hepatic lipid accumulation in darkness rats led us to explore the key genes involved in lipid metabolism that were regulated by *L. reuteri*. RNA sequencing on rat liver (*n* = 8/group) was thus performed because liver is the principal site of metabolism (Supplementary Data 1). We found 36 genes were highly expressed and 76 genes were lowly expressed (Fig. 2a, b) in the liver of darkness rats compared with both control rats and DL.reuteri rats, using differentially expressed gene (DEG) analysis. The Gene Ontology (GO) and Kyoto Encyclopedia of Genes and Genomes (KEGG) enrichments of these 112 (76 + 36) genes indicated TG catabolic process, response to glucocorticoid and insulin, circadian rhythm, peroxisome proliferator-activated receptor (PPAR) pathway, adenosine monophosphate-activated protein kinase (AMPK) pathway, NAFLD, etc. (Fig. 2c). We further performed gene set enrichment analysis (GSEA) to identify the altered biological functions and pathways in darkness rats (Supplementary Data 2). As expected, brown fat cell differentiation (*P* = 0.0045), TG catabolic process (*P* = 0.0481), response to glucocorticoid (*P* = 0.0073), and circadian rhythm (*P* < 0.001) were evidently downregulated in darkness rats comparing to control rats. We also observed that AMPK signaling pathway (*P* = 0.0041) and regulation of lipolysis in adipocyte (*P* = 0.0055) were prominently downregulated while PPAR signaling pathway (*P* = 0.0446) was upregulated in darkness rats. PPARs are fatty acid sensors regulating whole-body metabolism, and supraphysiological activation of PPARs causes obesity^25^. Hepatic AMPK plays essential roles in diminishing lipogenesis and enhancing lipolysis to attenuate dyslipidemia^26^. Moreover, we illustrated 17 key DEGs in GO and KEGG terms associated with lipid metabolism and circadian rhythm with a chord diagram (Fig. 2d), which might participate in the alleviation of *L. reuteri* on dyslipidemia in darkness rats. With weighted gene co-expression network analysis (WGCNA), a total of nine modules that contained functionally and biologically linked genes were identified. In these modules, the purple module was positively correlated with serum levels of CHOL (*r* = 0.4829, *P* = 0.0265), HDL (*r* = 0.4316, *P* = 0.0394), LDL (*r* = 0.4351, *P* = 0.0351), and NEFA (*r* = 0.4605, *P* = 0.0270), tested with Spearman’s correlation coefficients (Fig. 2e). GO and KEGG analyses of the genes of purple module also indicated cholesterol metabolism, circadian rhythm, glucocorticoid, insulin resistance, etc. (Fig. 2f).

Of 112 genes from DEG analysis with 39 genes from top enriched GO and KEGG terms related to metabolism and circadian rhythm of WGCNA-purple module, we identified 6 potentially *L. reuteri*-targeted hepatic genes involving *galanin receptor 1* (*Galr1)*, *nuclear receptors subfamily 1, group D, member 1 and 2* (*Nr1d1*, *Nr1d2*), *insulin induced gene 2* (*Insig2*), *sterol regulatory element binding transcription factor 1* (*Srebf1*), and *retinoid X receptor a* (*Rxra*) (Fig. 2g). GALR3 is expressed at moderate levels in discrete regions of the brain and at low levels in many central and peripheral tissues^27^. We also verified the low expression of *Galr3* in the liver tissue of rats and thus focused on the other two receptors. The mRNA expressions of *Galr1*, *Galr2*, *Nr1d1*, *Nr1d2*, *Insig2*, *Srebf1*, *liver X receptor a* (*Lxra*), and *Rxra* from qPCR analysis were similar to the results from RNA sequencing analysis (Fig. 2h).

In sequential sample collection of the constant darkness-induced PCOS-like rat model, 5 rats from each group were killed every 4 h from zeitgeber time (ZT)0 to ZT20 (Supplementary Fig. 1a). Body weight and adipocyte sizes remained similar between control and darkness rats (Supplementary Fig. 1b, c), but lipid dysmetabolism caused by darkness treatment was confirmed with Oil Red O staining, transmission electron microscopy as well as hepatic and serum lipid profiles (Supplementary Fig. 1d–g). Hepatic mRNA expression of *Nr1d1 Nr1d2*, *Srebf1*, *Insig2*, *Lxra*, *Rxra*, *Galr1*, and *Galr2* rhythmically differed over the course of a day between control and darkness rats (Supplementary Fig. 1h–p). Hence not only the expression level but also the expression rhythm of potential target genes in the liver altered due to circadian disruption.

### Increased GALR1 promotes SREBP1 expression via NR1D1, thus inducing lipid accumulation in the liver

**Fig. 3:**
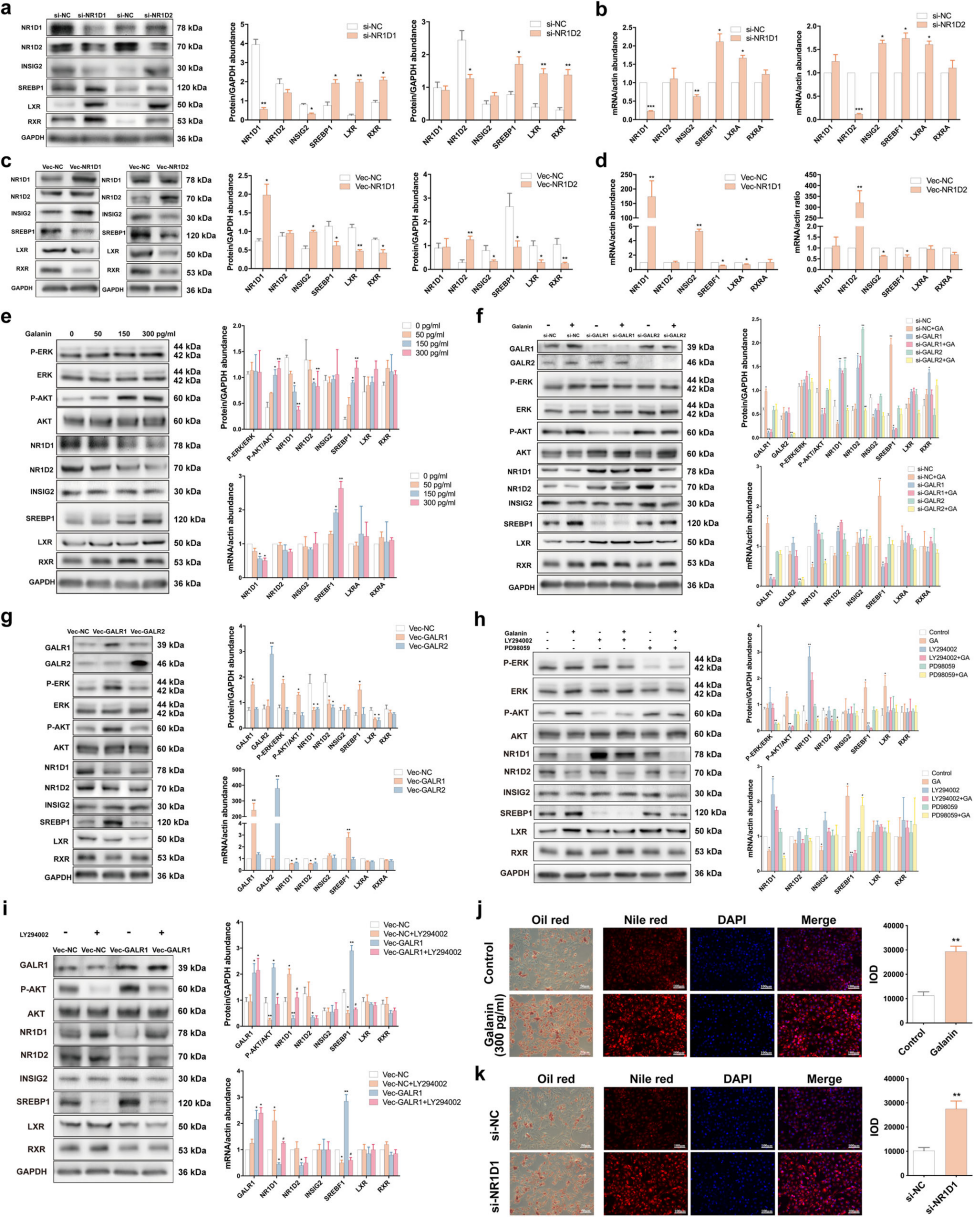
Increased GALR1 promotes SREBP1 expression via NR1D1, thus inducing lipid accumulation in HepG2 cells. mRNA and protein abundances of NR1D1, NR1D2, INSIG2, SREBP1, LXR, and RXR after *NR1D1* knockdown or *NR1D2* knockdown (**a**, **b**) as well as *NR1D1* overexpression or *NR1D2* overexpression (**c**, **d**) in HepG2 cells. **a**, **c** Left, representative images of western blot were shown. Right, immunoreactive bands were densitometrically quantified. **b**, **d** mRNA abundance detected by qPCR was presented. **e** mRNA and protein abundances of NR1D1, NR1D2, INSIG2, SREBP1, LXR, RXR, P-ERK, T-ERK, P-AKT, and T-AKT after galanin treatment (#1179, Tocris Bioscience, Bristol, UK) at the concentration of 0, 50, 150, and 300 pg/mL for 24 h in HepG2 cells. Left, representative images of western blot were shown. Right, immunoreactive bands were densitometrically quantified (above); mRNA abundance detected by qPCR was presented (below). **f** mRNA and protein abundances of GALR1, GALR2, NR1D1, NR1D2, INSIG2, SREBP1, LXR, RXR, P-ERK, T-ERK, P-AKT and T-AKT after *GALR1* knockdown or *GALR2* knockdown and further treatment with 300 pg/mL galanin for 24 h in HepG2 cells. **g** mRNA and protein abundances of GALR1, GALR2, NR1D1, NR1D2, INSIG2, SREBP1, LXR, RXR, P-ERK, T-ERK, P-AKT, and T-AKT after *GALR1* overexpression or *GALR2* overexpression in HepG2 cells. **h** mRNA and protein abundances of NR1D1, NR1D2, INSIG2, SREBP1, LXR, RXR, P-ERK, T-ERK, P-AKT, and T-AKT after treatment with(out) 300 pg/mL galanin, with(out) 10 μM LY294002 (#19-142, Sigma-Aldrich, St. Louis, USA) and with(out) 20 μM PD98059 (#19-143, Sigma-Aldrich). **i** mRNA and protein abundances of GALR1, NR1D1, NR1D2, INSIG2, SREBP1, LXR, RXR, P-AKT, and T-AKT after *GALR1* overexpression and further treatment with 10 μM LY294002 in HepG2 cells. GAPDH or *β-ACTIN* were used as loading controls for western blot and qPCR analyses. Representative Oil Red O staining and Nile Red staining after the induction of oleic acid and palmitic acid (OPA) and treatment with 300 pg/mL galanin for 24 h (**j**) or *NR1D1* siRNA (**k**) in HepG2 cells. Left, representative images were shown. Right, intensity was quantified. Blots and images are representative. Statistical analysis was performed with unpaired Student’s t-test or one-way ANOVA followed by Newman–Keuls multiple comparison test. Data present means ± SEM from 3 to 5 experiments. *P < 0.05, **P < 0.01, ***P < 0.001 against si-NC cells or against Vec-NC cells or against control cells; ^#^*P* < 0.05, ^##^*P* < 0.01 against si-GALR2 cells or against PD98059 cells or against Vec-GALR1 cells.

To probe possible specific molecular mechanisms underlying the contribution of target molecules to hepatic lipid metabolic abnormalities, we performed in vitro experiments with HepG2 cells. NR1D1 positively regulated INSIG2 and inhibited SREBP1, LXR, and RXR expression. Similar regulation of NR1D2 to SREBP1, LXR, and RXR was also detected (Fig. 3a–d). The addition of galanin to HepG2 cells distinctly increased phosphorylated protein kinase B (AKT) level and SREBP1 expression, and decreased NR1D1 and NR1D2 expression in a dose-dependent manner (Fig. 3e). Nevertheless, the treatment of HepG2 cells with *GALR1* siRNA, but not *GALR2* siRNA, inhibited the galanin’s ability to regulate phosphorylated AKT level and expression of NR1D1 and SREBP1 (Fig. 3f). The overexpression of *GALR1* or *GALR2* supported these results (Fig. 3g). Therefore, GALR1 might mediate the ability of galanin to regulate dyslipidemia. Besides, phosphoinositide 3-kinase (PI3K) inhibitor LY294002 impeded the regulation of galanin to NR1D1 and SREBP1 expression while ERK1/2 inhibitor PD98059 failed (Fig. 3h). LY294002 also hindered the effect of *GALR1* overexpression on NR1D1 and SREBP1 (Fig. 3i). Thus, GALR1 possibly regulated downstream NR1D1 and SREBP1 via the PI3K pathway. Moreover, galanin administration or *NR1D1* siRNA treatment in HepG2 cells apparently increased lipid accumulation (Fig. 3j, k). All these data indicate that increased GALR1 functions as a critical upstream factor in the PI3K/AKT pathway to suppress NR1D1 and promotes SREBP1 inducing hepatic lipid accumulation.

### Improvement in dyslipidemia of darkness rats by *L. reuteri* depends on GALR1 signaling

**Fig. 4:**
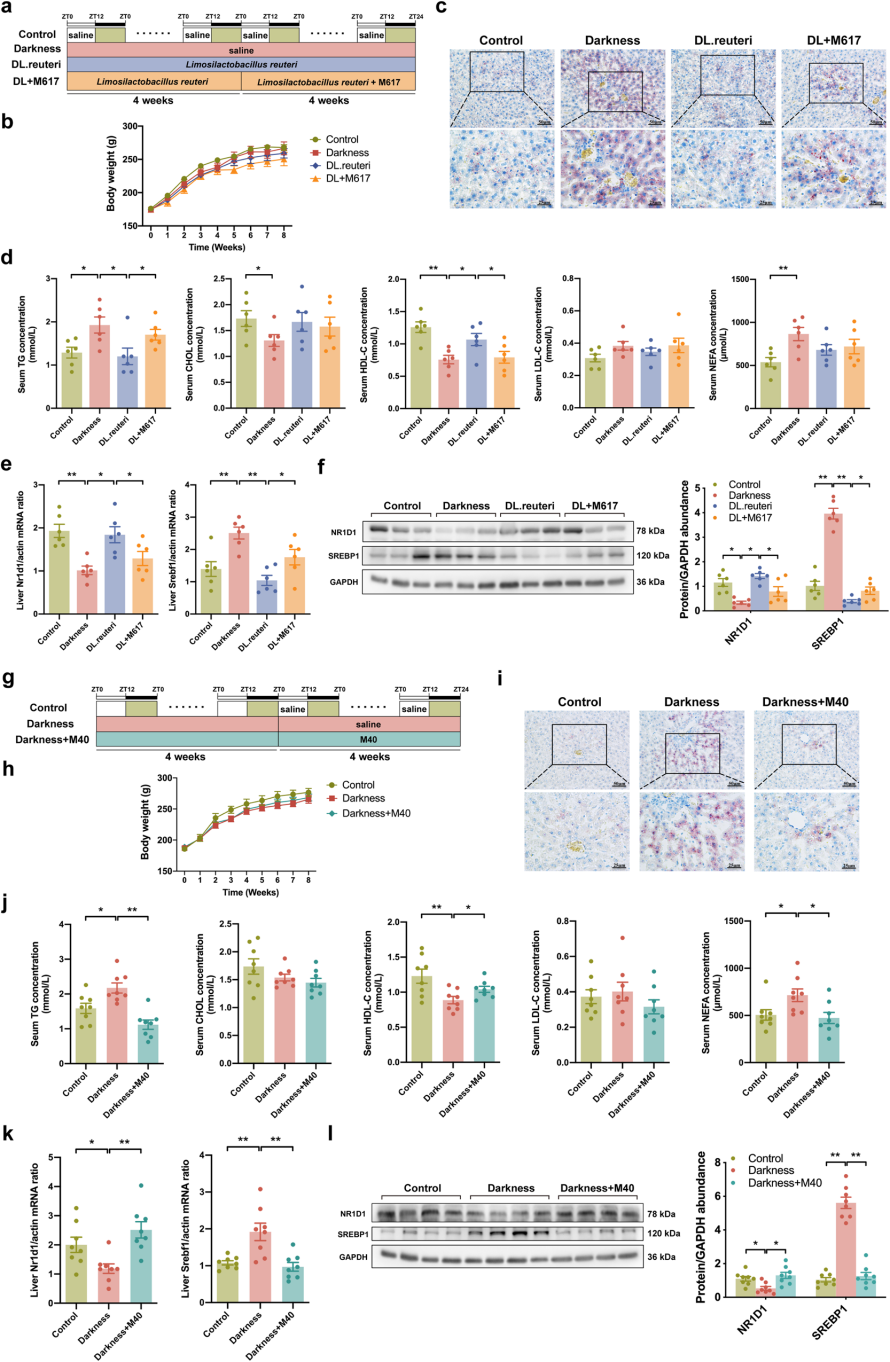
GALR1 mediates the improvement in dyslipidemia of darkness rats by *L. reuteri*. **a** Timeline depicting the treatments of darkness, *L. reuteri*, and M617 in different groups of the M617-treated rat model (*n* = 6 per group). **b** Body weight changes. **c** Representative Oil Red O staining of liver. Scale bar: 50 μm and 25 μm. **d** Left to right, serum concentrations of TG, CHOL, HDL-C, LDL-C, and NEFA detected by ELISA. **e** mRNA abundances of *Nr1d1* and *Srebf1* in rat liver. **f** Protein abundances of NR1D1 and SREBP1 in rat liver. Left, representative images of western blot were shown. Right, immunoreactive bands were densitometrically quantified. **g** Timeline depicting the treatments of darkness and M40 in different groups of the M40-treated rat model (*n* = 8 per group). **h** Body weight changes. **i** Representative Oil Red O staining of liver. Scale bar: 50 μm and 25 μm. **j** Left to right, serum concentrations of TG, CHOL, HDL-C, LDL-C, and NEFA detected by ELISA. **k** mRNA abundances of *Nr1d1* and *Srebf1* in rat liver. **l** Protein abundances of NR1D1 and SREBP1 in rat liver. Left, representative images of western blot were shown. Right, immunoreactive bands were densitometrically quantified. GAPDH or *β-Actin* were used as loading controls for western blot and qPCR analyses. Statistical analysis was performed with one-way ANOVA followed by Newman–Keuls multiple comparison test. Data present means ± SEM. *P < 0.05, **P < 0.01.

GALR1 agonist M617 was used to certify the vital role of GALR1 in the alleviation of *L. reuteri* on dyslipidemia (Fig. 4a). The body weights did not significantly change among the four groups (Fig. 4b). Nevertheless, M617 administration impeded the amelioration of *L. reuteri* on hepatic lipid accumulation in darkness rats (Fig. 4c). Upon analyzing the serum lipid profiles, rats that received both M617 and *L. reuteri* treatments demonstrated increased TG level and decreased HDL-C level compared with *L. reuteri*-treated rats (Fig. 4d). Meanwhile, M617 inhibited the high expression of NR1D1, and promoted the low expression of SREBP1 in DL.reuteri rats (Fig. 4e, f). That’s to say, hepatic GALR1-NR1D1-SREBP1 pathway might mediate the amelioration of *L. reuteri* supplementation on dyslipidemia in darkness rats.

On the other hand, GALRs antagonist M40 was used to verify the role of galanin-GALR1 in darkness-induced dyslipidemia (Fig. 4g). The body weights remained similar among the three groups (Fig. 4h). Besides reducing the lipid accumulation in the liver of darkness rats (Fig. 4i), M40 treatment moderated the levels of serum TG, HDL-C, and NEFA (Fig. 4j). As regards potential hepatic downstream signaling molecules, M40 increased the expression of NR1D1 and decreased the expression of SREBP1 which were initially low and high respectively in darkness rats (Fig. 4k, l). Since the serum galanin concentration that was elevated in darkness rats remained after *L. reuteri* treatment (Fig. 2i and Supplementary Fig. 1q), we posit that the modulation by *L. reuteri* regimen directly depends on GALR1 signaling.

### *L. reuteri* restructures gut microbiota and metabolites in darkness rats

**Fig. 5:**
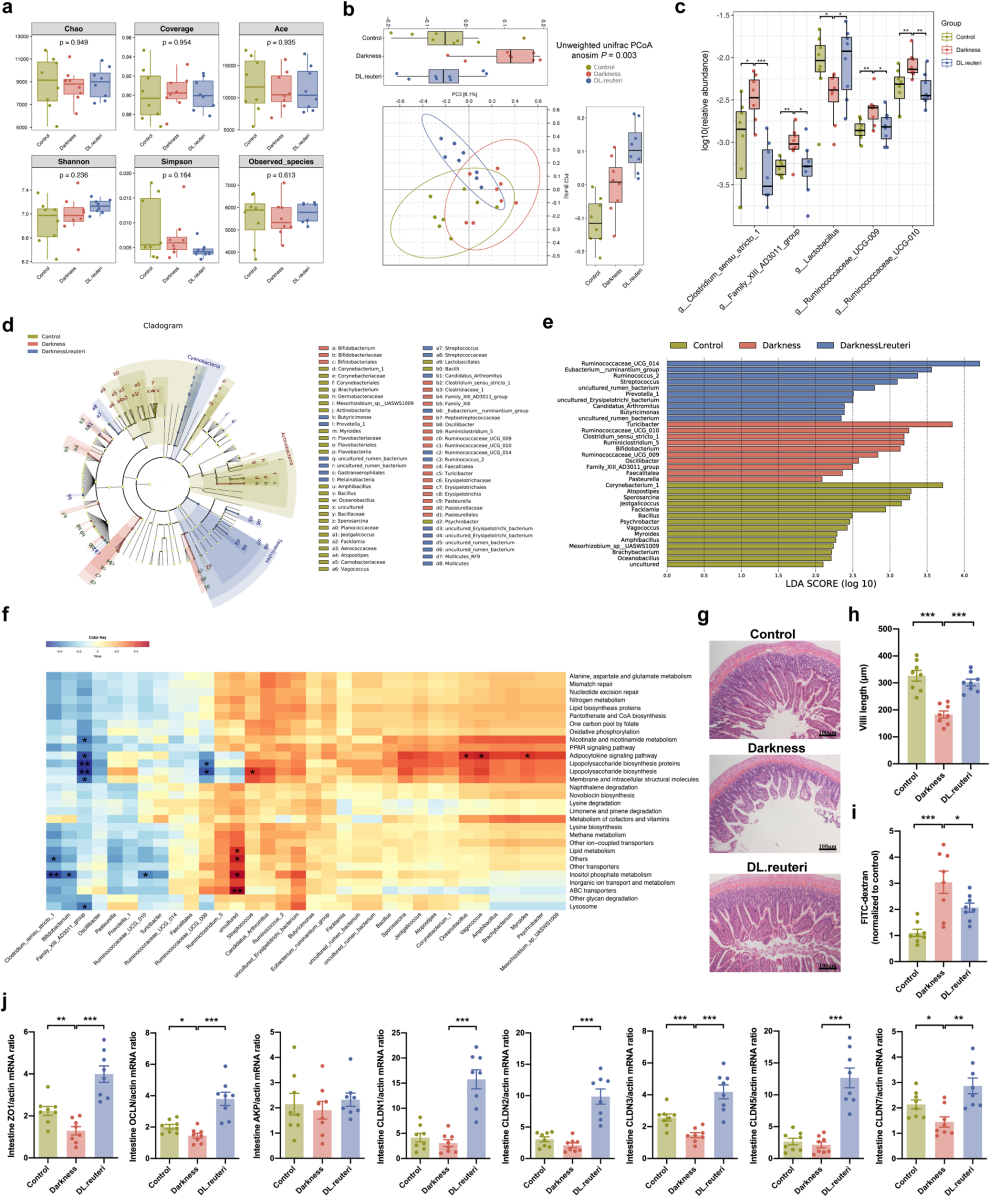
Faecal microbiome analysis of *L. reuteri*-treated darkness rats. **a** Boxplots of a-diversity calculated by Chao, Coverage, Ace, Shannon, Simpson, and Observed species. **b** PCoA plot based on unweighted Unifrac distance was used to test the variations of microbial communities among groups. Analysis of similarities (anosim): *P* = 0.003 with 999 permutations. **c** Relative abundance of differential genera (highly or lowly expressed in darkness rats compared with control and DL.reuteri rats, *P* < 0.05), calculated with Kruskal–Wallis test followed by Dunn’s multiple comparison test. **d** Cladogram of LDA effect size analysis. Microbiota with no significant difference were stained into yellow, taxonomic biomarkers were stained following the group category. **e** Genera biomarkers within groups identified by LDA effect size analysis with an LDA score > 2. **f** Prediction of functional potential with PICRUSt2.0 analysis using the above genera biomarkers (*P < 0.05, **P < 0.01). **g** Representative hematoxylin and eosin staining of ileum. Scale bar: 100 μm. **h** Villi length of ileum was measured. **i** Gut permeability was tested. Fluorescence intensity in sera of each sample was normalized to control group average. **j** Left to right, mRNA abundances of *Zo1*, *Ocln*, *Akp*, *Cldn1*, *Cldn2*, *Cldn3*, *Cldn5*, and *Cldn7* in rat ileum. *β-Actin* was used as a loading control for qPCR analyses. Statistical analysis (**h**–**j**) was performed with one-way ANOVA followed by Newman–Keuls multiple comparison test. The boxplot elements were defined as following: center line, median; box limits, upper, and lower quartiles; whiskers, 1.5 × interquartile range. Points outside the whiskers represented outlier samples. *n* = 8 per group. Data present means ± SEM. *P < 0.05, **P < 0.01, ***P < 0.001.

The above results gave rise to the investigation of signaling mediators between *L. reuteri* and hepatic GALR1-NR1D1-SREBP1 pathway. By functioning as a probiotic, the impact of *L. reuteri* on host metabolism is intestinal flora dependent. Gut microbiota-driven metabolites are implicated as potential mediators in the interaction between microbiome communities and energy homeostasis^28–31^. Therefore, we propose that gut microbiota and metabolites might mediate the effect of *L. reuteri* on hepatic lipid dysmetabolism. 16 S rRNA gene sequencing analysis of faecal bacteria isolated from control, darkness, and DL.reuteri rats (*n* = 8/group) was used to determine structural changes in gut microbiota in response to *L. reuteri* (Supplementary Data 3). The microbiome α diversity of three groups, presented with Chao, Coverage, Ace, Shannon, Simpson, and Observed species analyses remained at the similar level (Fig. 5a). The unweighted Unifrac distance of DL.reuteri rats was different from other groups (anoism *P* = 0.003), which indicated that *L. reuteri* distinctly changed β diversity (Fig. 5b). *L. reuteri* totally overturned the influence of darkness on rats with increased *Lactobacillus* and decreased *Clostridium sensu stricto 1*, *Family XIII AD3011 group*, *Ruminococcaceae UCG-009*, and *Ruminococcaceae UCG-010* at the genus level (Fig. 5c and Supplementary Data 4). To infer the putative role of gut microbiota in *L. reuteri*-treated darkness rats, 34 represented genera (14 from control, 10 from darkness, and 10 from DL.reuteri rats) were employed to predict the functional composition. These flora were identified by Linear discriminant analysis (LDA) effect size (LEfSe) (Fig. 5d, e). Metagenomic inference showed that five differentially expressed genera might interact with genes referring to nicotinate and nicotinamide metabolism, adipocytokine signaling pathway, lipopolysaccharide biosynthesis, inositol phosphate metabolism, etc. (Fig. 5f), most of which are closely related to lipid metabolism.

The integrity of the intestinal barrier is critical in the interaction between microbiota and metabolites in the gastrointestinal tract and the systemic metabolism^29^. We found that the ileum villi were flattened with shortened lengths in darkness rats; however, this effect was ameliorated by *L. reuteri* (Fig. 5g, h). Fluorescein isothiocyanate (FITC)–dextran assay indicated that darkness rats receiving *L. reuteri* showed reduced gut permeability compared with darkness rats (Fig. 5i). The administration of *L. reuteri* to darkness rats also elevated several ileal tight-junction genes that were decreased in darkness rats, especially *zonula occludens 1* (*Zo1*), *occludin* (*Ocln*), *claudin* (*Cldn*) *3*, and *Cldn7* (Fig. 5j). Hence, these results imply the benefits of administering *L. reuteri* on the intestinal barrier that has been damaged by darkness exposure. This connects alterations in intestinal microbiota and metabolites to changes in circulating metabolites.

**Fig. 6:**
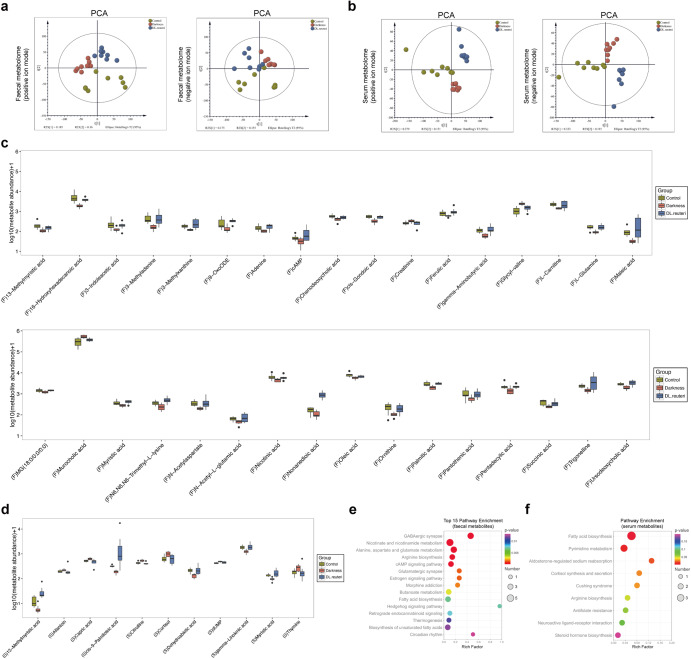
Faecal and serum metabolomes analyses of *L. reuteri*-treated darkness rats. Scatter plot of scores from PCA of faecal metabolome (**a**) and serum metabolome (**b**) in positive ion and negative ion modes. Relative abundances of differential metabolites (highly or lowly expressed in darkness rats compared with control and DL.reuteri rats) identified by analyses of faecal metabolome (**c**) and serum metabolome (**d**). Based on the OPLS-DA model, variable importance in the projection (VIP) value > 1. *P* < 0.05 by unpaired Student’s t-test of the peak area between each two groups. Predictive KEGG pathway analysis performed on the MetaboAnalyst website using the above 33 differential faecal metabolites (**e**) and 11 differential serum metabolites (**f**). The boxplot elements were defined as following: center line, median; box limits, upper, and lower quartiles; whiskers, 1.5 × interquartile range. Points outside the whiskers represented outlier samples. *n* = 8 per group.

We further detected the untargeted metabolite profiling of both faecal samples (Supplementary Data 5) and serum samples (Supplementary Data 6) from control, darkness, and DL.reuteri rats (*n* = 8/group), which revealed marked group differences by principal component analysis (PCA), partial least squares discrimination analysis (PLS-DA), and orthogonal PLS-DA (OPLS-DA) (Fig. 6a, b and Supplementary Fig. 2a–d). *L. reuteri* reversed the altered abundance of metabolites in darkness rats including 33 faecal metabolites (Fig. 6c and Supplementary Data 7) and 11 serum metabolites (Fig. 6d and Supplementary Data 7). The predictive pathway enrichment was performed using differential metabolites. Both faecal and serum differential metabolites illustrated amino acid metabolism and lipid metabolism including fatty acid biosynthesis, biosynthesis of unsaturated fatty acids, etc. (Fig. 6e, f). Combining microbial functional prediction with that of metabolomes showed that lipid metabolism was specifically enriched in the benefits of *L. reuteri* regimen in darkness rats. That is to say, gut microbiota and metabolites indeed mediate the regulation of *L. reuteri* to lipid dysmetabolism in darkness rats.

### *L. reuteri* decreases capric acid and alleviates dyslipidemia via GALR1 signaling in darkness rats

**Fig. 7:**
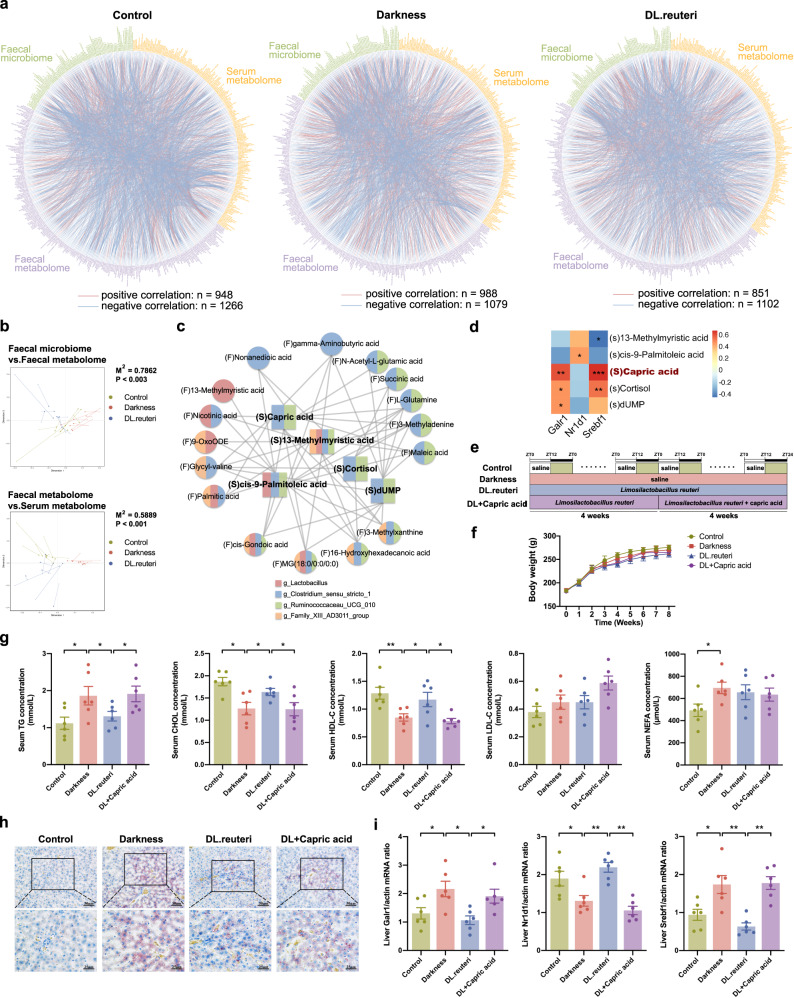
*L. reuteri* decreases capric acid and alleviates dyslipidemia via GALR1 signaling in darkness rats. **a** Multiomics correlation networks of all variables for the faecal microbiome (green), faecal metabolome (purple) and serum metabolome (yellow) within control rats (left), darkness rats (middle), and DL.reuteri rats (right). Vertices indicate omics variables, and lines indicate a significant Spearman’s rank correlation coefficient at |ρ | > 0.8 and *P* < 0.05. Red connections indicate positive correlation, and blue connections show negative correlations. **b** Procrustes analyses of faecal microbiome versus faecal metabolome (above) and of faecal metabolome versus serum metabolome (below). **c** Spearman correlation network between target faecal metabolites (circle) and target serum metabolites (square) (*P* < 0.05). The color of each metabolite was determined by their correlations with target genera (*P* < 0.05): Pink, *Lactobacillus*; Blue, *Clostridium sensu stricto 1*; Green, *Ruminococcaceae UCG-010*; Yellow, *Family XIII AD3011 group*. **d** Spearman rank correlations between target serum metabolites and mRNA expression of *Galr1*, *Nr1d1*, and *Srebf1* in rat liver (*P < 0.05, **P < 0.01, ***P < 0.001). **e** Timeline depicting the treatments of darkness, *L. reuteri*, and capric acid in different groups of the capric acid-treated rat model (*n* = 6 per group). **f** Body weight changes. **g** Left to right, serum concentrations of TG, CHOL, HDL-C, LDL-C, and NEFA detected by ELISA. **h** Representative Oil Red O staining of liver. Scale bar: 50 μm and 25 μm. **i** mRNA abundances of *Galr1*, *Nr1d1,* and *Srebf1* in rat liver. *β-Actin* was used as a loading control for qPCR analyses. Statistical analysis (**g**, **i**) was performed with one-way ANOVA followed by Newman–Keuls multiple comparison test. Data present means ± SEM. *P < 0.05, **P < 0.01.

Having identified microbiome and metabolome signatures that were upregulated or downregulated by *L. reuteri* in darkness rats, the next step was to assess the interplay across gut microbiota compositions, faecal metabolites, serum metabolites, and hepatic GALR1-mediated signaling pathway, in relation to the observed impact of *L. reuteri* regimen on dyslipidemia in darkness rats. We carried out multiomics Spearman correlation analysis of all enriched gut genera, faecal, and serum metabolites within each group. Tight but different connections were certified in control, darkness, and DL.reuteri rats (|ρ | > 0.8 and *P* < 0.05) (Fig. 7a). Only 2.4% of correlations were common in control and darkness rats (Supplementary Fig. 3a). Meanwhile, 57 common correlations between control and DL.reuteri rats principally predicted amino acid metabolism and lipid metabolism, which also supported the observation that *L. reuteri* regimen reduced the dyslipidemia of darkness rats (Supplementary Fig. 3b, c). Moreover, procrustes analysis presented a strong cooperativity of faecal microbiome and faecal metabolome (*P* < 0.003), as well as that of faecal metabolome and serum metabolome (*P* < 0.001), indicating the contribution of modified gut microbiota to metabolic alterations in intestinal tract and serum after *L. reuteri* treatment in darkness rats (Fig. 7b). Multiple Spearman’s rank correlation coefficients were then calculated using the above differentially expressed 5 genera, 33 faecal metabolites, 11 serum metabolites, and serum lipid parameters to identify key metabolites involved in the *L. reuteri*-alleviation of dyslipidemia in darkness rats (Supplementary Fig. 4a–h). Altered abundances of *Lactobacillus*, *Clostridium sensu stricto 1*, *Ruminococcaceae UCG-010*, and *Family XIII AD3011 group* caused by the *L. reuteri* regimen were closely associated to changes in multiple faecal metabolites as well as serum metabolites including cortisol, cis-9-palmitoleic acid, 13-methylmyristic acid, capric acid, and deoxyuridine monophosphate (dUMP), subsequently related to modulation of dyslipidemia (Fig. 7c). Because the essential role of corticosterone in the stress of rodents has been established^32^, we reassessed the serum concentrations of cortisol and corticosterone in rats using enzyme-linked immunosorbent assay (ELISA) kits, results of which were consistent to the metabolome data (Supplementary Fig. 4i). Besides of the close collaboration between targeted gut microbiota and metabolites, strong correlations within genera as well as correlations within metabolites were also observed (Supplementary Fig. 4j–l), thus constructing a complex interacting network.

To further determine the key serum metabolites that mediate the regulation of GALR1 by *L. reuteri* and improve lipids, we calculated the correlations between targeted serum metabolites and hepatic mRNA expression of target genes. The strong associations of *Galr1* expression with capric acid, cortisol, and dUMP were indicated (Fig. 7d). Since cortisol is inactive in rodents, we next verified the in vivo role of capric acid as a mediator of the beneficial effects on GALR1 signaling induced by *L. reuteri* (Fig. 7e). There was no obvious difference in body weight among the four groups (Fig. 7f). Consistent with the above data, *L. reuteri* attenuated the high level of TG and low levels of CHOL and HDL-C in the sera of darkness rats. However, this improvement was reversed by capric acid supplementation (Fig. 7g). Meanwhile, capric acid facilitated the accumulation of hepatic lipids in which were initially low in DL.reuteri rats as shown by Oil Red O staining (Fig. 7h). Of note, capric acid treatment evidently restrained the inhibiting effect of *L. reuteri* on the expression of GALR1 and SREBP1 as well as its promoting effect on the expression of NR1D1 in the liver of darkness rats (Fig. 7i). Accordingly, capric acid takes an important role in the amelioration of GALR1-regulated lipid metabolism by *L. reuteri*. In other words, *L. reuteri* supplementation exerts the protective effect against constant darkness-induced lipid dysmetabolism of PCOS partly through caprid acid and further hepatic GALR1 signaling pathway.

## Discussion

**Fig. 8:**
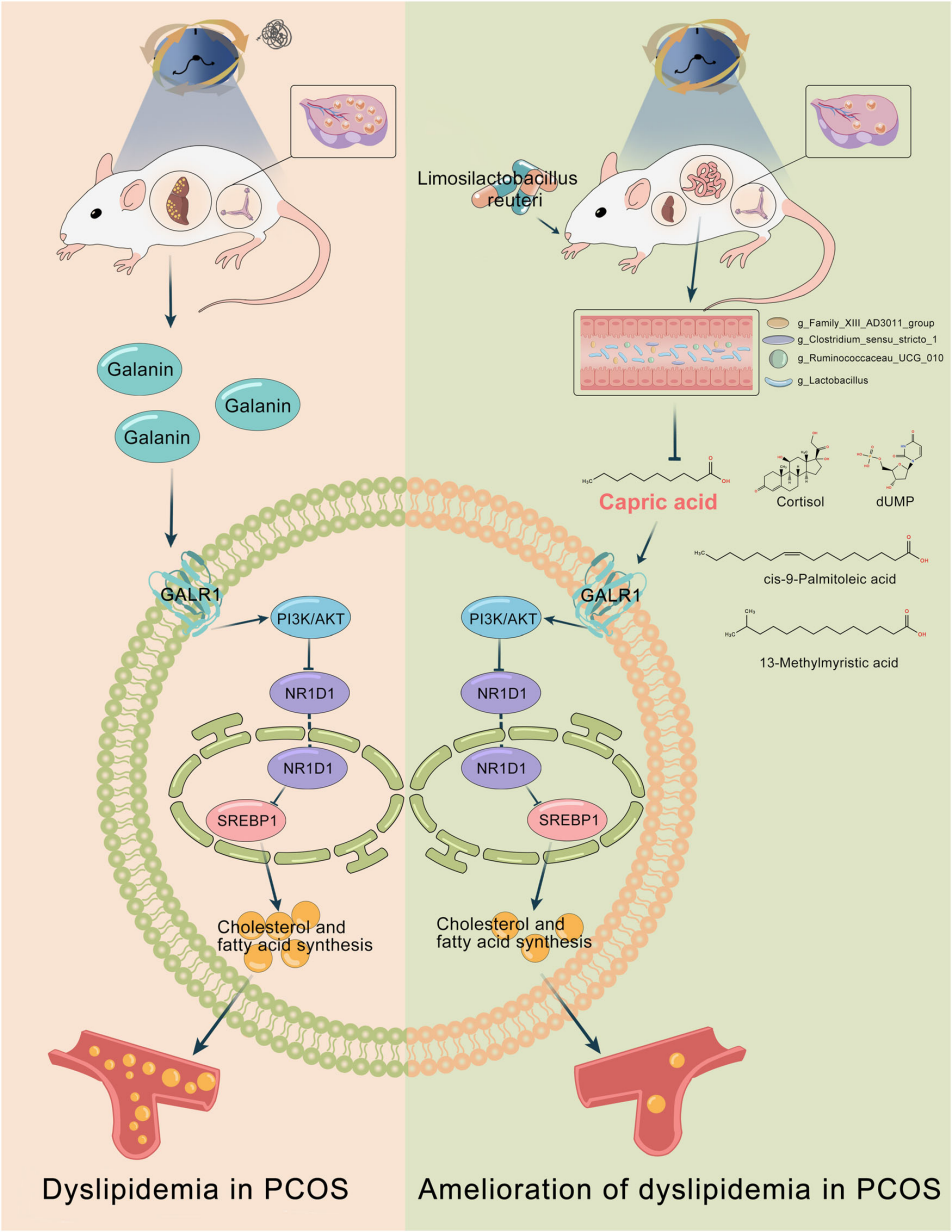
Proposed mechanisms for the amelioration of *L. reuteri* on dyslipidemia in circadian dysrhythmia-induced PCOS-like rats. Left, circadian dysrhythmia due to constant darkness resulted in dyslipidemia and reproductive hallmarks of PCOS in rats. Elevated galanin-GALR1 induced by darkness exposure functioned as an upstream factor of PI3K/AKT pathway and further suppressed NR1D1-induced *SREBF1* transcription and translation, thus inducing hepatic lipid accumulation in PCOS-like rats. Right, *L. reuteri* supplementation ameliorated dyslipidemia and reproductive hallmarks in circadian dysrhythmia-induced PCOS-like rats. *L. reuteri* restructured microbiome-metabolome network in darkness rats ameliorating the abundance of *Lactobacillus*, *Clostridium sensu stricto 1*, *Ruminococcaceae UCG-010*, and *Family XIII AD3011 group*, followed by varied serum levels of cortisol, cis-9-palmitoleic acid, 13-methylmyristic acid, capric acid, and dUMP. Notably, capric acid mediated the inhibition of *L. reuteri* on hepatic GALR1-PI3K/AKT-NR1D1-SREBP1 pathway, which eventually alleviated dyslipidemia.

Emerging evidence has implicated the intersection between the circadian rhythm and gut microbiota with the host metabolism^28^; however, little is known concerning the mechanisms underlying this intersection especially in women with PCOS. Our study identifies the lipid disorder in circadian disruption-induced PCOS-like rats. We figure out for the first time that a restructured microbiome-metabolome network following the dietary administration of *L. reuteri* could provide substantial protection against dyslipidemia in constant darkness rats. Notably, *L. reuteri* exerts the protective effect on lipid homeostasis partly through caprid acid and further hepatic GALR1-NR1D1-SREBP1 signaling pathway (Fig. 8). Our data thus suggest that *L. reuteri*–capric acid–GALR1 axis could have tremendous potential for preventing and treating lipid dysmetabolism associated with biorhythm disorders, especially PCOS.

We observed that water intake and food consumption remained consistent between control and darkness rats at the end of the treatment (Supplementary Fig. 5a, b). In addition to dyslipidemia, increased fasting blood glucose and impaired glucose tolerance were also found in darkness rats (Supplementary Fig. 5c, d). However, glucolipid metabolic dysregulation in the host might be not enough to cause significant weight gain in darkness rats despite the fact that weight gain is a key hallmark of PCOS. The normal chow diet we fed rats might take a role. A previous study presents that high-fat diet and light schedule show interaction effects to increase body mass while there are no individual effects of light schedule present in mice fed a low-fat diet^33^. A suitable animal model mimicking all features of PCOS in humans is still being identified.

The endogenous galanin-GALRs system has a special role in adapting food intake, glucose homeostasis, adipogenesis, arousal or sleep, neuroendocrinology, etc.^34^. Central galanin neurons promote sleep in mice and a decline in galanin neurons might lead to sleep fragmentation^35,36^. Other than our observation identifying GALR1 as an essential regulator in peripheral circadian clock, more evidence is required in the future to uncover its role in systemic circadian rhythm. Additionally, serum galanin level is positively associated with HbA1c level in diabetes mellitus and correlated with insulin resistance and TG level in obese people^37,38^. GALR1 is reported to mediate the obesogenic effects of galanin^37^; however, the functional role of hepatic GALR1 and the impact of galanin-GALRs on host metabolism are still controversial. For example, the increase of blood glucose by peripheral treatment of the GALR1-specific agonist is in contrast to the suppression role of central galanin administration^37^. Our results also illustrated GALR1 antagonist (M40) as a potential therapeutic target for dyslipidemia in circadian disruption-ignited PCOS. Nevertheless, another study portrays the protective effects of galanin injection on inflammatory, hormonal, and glucose parameters in the PCOS-like rat model following a single injection of estradiol valerate^39^. Although the seemingly paradoxical conclusion could be attributed to the different animal models used, intricate mechanisms underlying the action of galanin-GALRs in metabolism and PCOS remain unexplored.

Consistent with our results, mounting evidences have also characterized hepatic *Nr1d1* deletion mice and *Nr1d1*^−/−^ mice with lipid disturbances probably through regulation of SREBP1 and INSIG2^40,41^. Intriguingly, a recent study elucidates the attenuation effect of systemic NR1D1 agonist administration on hepatic steatosis and inflammation in non-alcoholic steatohepatitis (NASH) mice might be attributed to the enhancement of gut barrier^42^. An ileal transcriptome has been detected in *Lactobacillus*-treated healthy mice and the circadian rhythm pathway presents maximum enrichment^43^. We also observed the improvement of *L. reuteri* on intestinal barrier integrity in darkness rats. Meanwhile, *L. reuteri* dramatically increased *Nr1d1* and *Nr1d2* expression in the intestine of darkness rats, though only *Nr1d1* expression differed between control and darkness rats (Supplementary Fig. 6a, b). Thus, we inferred that ileal NR1D1 might play a partial role in the modulation of *L. reuteri* on darkness-induced gut barrier dysfunction and dyslipidemia. Special attention should be focused on the circadian rhythm of gut itself.

*L. reuteri* supplement reduced the abundance of *Clostridium sensu stricto 1*, *Family XIII AD3011 group*, *Ruminococcaceae UCG-009*, and *Ruminococcaceae UCG-010* in darkness rats. Consistent with our findings, a previous study using constant darkness mice also shows a high abundance of *Clostridia* in the small intestine^44^. The genus *Clostridium sensu stricto* is markedly increased in mice with NASH^45^, and it positively correlates with the waistline measurements, body mass index as well as LDL, TG, and CHOL levels in adults^46^. The family *Ruminococcaceae* and *Family XIII AD3011* are associated with PCOS^47^. Furthermore, *Ruminococcaceae* UCG-010 and *Ruminococcaceae* UCG-009 are significantly positively correlated with plasma concentration of trimethylamine-N-oxide^48^, which promotes the development of atherosclerosis in mice. Thus, high levels of the above four genera might facilitate the metabolic abnormalities and even hormone imbalance in darkness rats. Their specific functional actions in circadian disruption-derived PCOS are well worthy of investigations. What’s more, we also performed 16 S rRNA gene sequencing analysis of faecal microbiome in control, darkness, and darkness+M40 rats (*n* = 5/group) (Supplementary Data 8). The microbiome α diversity remained at the similar level in three groups, while the β diversity was distinctly changed (anoism *P* = 0.006) (Supplementary Fig. 7a, b). As shown, M40 treatment did not alleviate the high abundances of *Clostridium sensu stricto 1*, *Family XIII AD3011 group*, *Ruminococcaceae UCG-009*, and *Ruminococcaceae UCG-010* in darkness rats (Supplementary Fig. 7c). Unexpectedly, the low abundance of *Lactobacillus* in darkness rats was still substantially reversed by M40 injection. Besides, the low abundances of *Anaeroplasma*, *Jeotgalicoccus*, and *Turicibacter* as well as high abundances of *Caldicoprobacter* and *GCA-900066575* in darkness rats were also moderated (Supplementary Fig. 7d). Predictive function analysis portrayed the potential roles of M40 treatment in fatty acid metabolism, steroid biosynthesis, circadian entrainment in darkness rats, which supported the current posit of *L. reuteri* administration in darkness rats (Supplementary Fig. 7e).

Capric acid is a major component of medium chain triglycerides of ubiquitous dietary origin. As a medium-chain fatty acid, dietary capric acid is directly transferred to the liver via the portal vein and rapidly metabolized through the bile acid metabolism pathway^49^. Our findings revealed the increased level of serum capric acid as well as decreased levels of faecal chenodeoxycholic acid (CDCA) and ursodeoxycholic acid (UDCA) in darkness rats compared with controls. Particularly, the level of capric acid and the levels of these two bile acids were distinctly negatively correlated (Supplementary Fig. 4h), indicating that the hepatic metabolization of capric acid might be inhibited in darkness rats. Excessive capric acid was supposed to stimulate GALR1-NR1D1-SREBP1 pathway giving rise to hepatic lipid accumulation. Nevertheless, the level of serum capric acid was obviously reduced and the levels of faecal CDCA, UDCA, and tauroursodeoxycholic acid were apparently elevated in darkness rats receiving *L. reuteri* supplementation. Meanwhile, the low abundance of *Lactobacillus*, reportedly showing bile salt hydrolase activity^50^, in darkness rats was also increased by *L. reuteri* treatment. Accordingly, we suggest that the catabolism of capric acid might be enhanced by *L. reuteri* and the activated GALR1 signaling due to capric acid was thus restrained in DL.reuteri rats, contributing to the amelioration of dyslipidemia. A previous study illustrates that supplementation with capric acid and its triglyceride form improves glucose sensitivity and lipid profiles in diabetic mice^51^. The promising role of capric acid in PCOS is also elaborated that capric acid treatment inhibits androgen biosynthesis and reverses insulin resistance in letrozole-induced PCOS-like rats^52^. Conversely, our study presented that capric acid administration encumbered the alleviation of *L. reuteri* on constant darkness-induced dyslipidemia of PCOS-like rats, thus shedding light on a novel mechanism accounting for the intricate effect of capric acid on lipids. Interestingly, capric acid is previously illustrated as a PPARγ activator^51^, and the PPAR signaling pathway was remarkably indicated in our KEGG analysis of hepatic DEGs profiles in *L. reuteri*-treated darkness rats. As the heterodimerization partner of PPARγ, high expression of RXR in darkness rats also descended due to *L. reuteri* administration. Although PPAR ligands such as thiazolidinediones can reduce insulin resistance and hyperglycemia, supraphysiological activation of PPAR causes obesity^25^. Growing evidence demonstrates that appropriate functional antagonism of PPARγ/RXR ameliorates fatty liver, which may be a logical approach to protection against obesity and related diseases^25,53,54^. Therefore, declined capric acid due to *L. reuteri* supplementation is also speculated to inactivate PPARγ/RXR besides of GALR1 signaling, contributing to the decrease of lipid contents in the liver of darkness rats. More research studies are still needed to comprehensively uncover the complicated role of capric acid in metabolic disorders, particularly associated with circadian dysrhythmia.

Although there are gaps of knowledge regarding the functional role of 13-methylmyristic acid in metabolism and PCOS, higher serum cortisol level is observed in the serum of women with PCOS^55,56^. The combination of high levels of melatonin and cortisol in the morning in PCOS is possibly a sign of disrupted circadian biorhythm^55^. Still, conflicting information exists regarding the interaction between palmitoleic acid and host metabolism. Palmitoleic acid restores glucose intolerance, promotes weight loss, and reduces inflammation in cell experiments, animal models, and even clinical trials; however, the increased level of palmitoleic acid in obese patients implies a higher risk of metabolic syndrome^57^. Their crucial roles in the amelioration of *L. reuteri* on dyslipidemia in darkness rats merit further investigations. As a matter of fact, short-chain fatty acids (SCFAs) and bile acids are considered as two main groups of microbe-derived metabolites that link gut microbiota to host metabolic outcomes and in turn influence host circadian rhythms^28,30^. The major SCFAs, butyrate, propionate, and acetate, were not observed in our target metabolome network. But the butyrate producers (family *Clostridiaceae* and family *Ruminococcaceae*), and the vital intermediary in microbial propionate synthesis (succinic acid)^58^ were enriched in the network regulated by *L. reuteri*. Bile acids exert metabolic functions by serving as highly efficacious farnesoid X receptor ligands or antagonists that participate in glucose and lipid homeostasis, immune responses, and insulin signaling^50^. As mentioned above, CDCA, UDCA, and murocholic acid were rescued by *L. reuteri* regimen in darkness rats. Although the restoration of these bile acids levels was not correlated with darkness-induced dyslipidemia, they might be closely related to other phenotypes caused by circadian disruption. Similarly, several well-known microbes-driven metabolites were also enriched in the modulation of *L. reuteri* regimen (i.e., L-carnitine, 3-indoleacetic acid, γ-linolenic acid, dehydroabietic acid, etc.) but were not associated with dyslipidemia. Their prospective roles in PCOS and biorhythm disorders ought to be addressed in the future.

In conclusion, our results propose that dietary supplementation of *L. reuteri* can protect against circadian dysrhythmia-induced PCOS by improving lipid dysmetabolism. *L. reuteri* decreases the enrichment of *Clostridium sensu stricto 1* and *Ruminococcaceae UCG-010* as well as microbiota-derived capric acid, which further inhibits hepatic GALR1-NR1D1-SREBP1 pathway activation and contributes to the amelioration of dyslipidemia. This study highlights the importance of *L. reuteri*–capric acid–GALR1 axis in the maintenance of lipid homeostasis, which may serve as an efficacious target in the treatment of biorhythm disorder-ignited dyslipidemia in PCOS.

## Methods

### Animal experiments

Female Sprague–Dawley rats of 6-week-old (Vital River Laboratory Animal Technology, Beijing, China) were housed in an animal lab under specific pathogen free conditions with three or four rats per cage. All rats were kept at a temperature of 21°C ± 2°C and a humidity of 65% ± 5 %. They received normal chow diet (#M02-F, Shanghai SLAC Laboratory Animal Co., Ltd, Shanghai, China) and autoclaved, sterilized water they could freely access ad libitum. The composition of this diet was 9.7% water, 20.5% protein, 4.62% fat, 4.35% cellulose, 6.2% crude ash, 1.24% calcium, 0.91% phosphorus, 1.3% lysine, 0.68% methionine+cystine, etc. After 8-week treatment, all rats were left to fast for 16 h (5 p.m. to 9 a.m.) with free access to water. Then faecal samples were collected before sacrifice, which were ready for 16 S rRNA gene sequencing and untargeted metabolomics. Next, serum samples were collected for untargeted metabolomics and other parameter detection. Meanwhile, tissues samples were collected and stored at −80 °C or fixed with 4% paraformaldehyde for further experiments. All animal experimental procedures were reviewed and approved by the Committee on Laboratory Animal Research of Shanghai Jiao Tong University, China. Animal welfare and experimental protocols were strictly in accordance with the guidelines for the care and use of laboratory animals.

### Establishment of *L. reuteri*-treated rat model

Rats were randomly divided into three groups namely: control, darkness, and DL.reuteri groups (*n* = 8/group). The control group received oral saline once a day and dwelt housed under in a 12:12 h light-dark cycle (lights on at 7:30 a.m. and off at 7:30 p.m.). The darkness group and DL.reuteri group were fed on saline or *L. reuteri* (10^10^ CFU/mL, 100 mg/d) (Taiwan Yaxin Biotechnology, Taiwan, China), respectively, by daily oral gavage in constant darkness for an 8-weeks’ period^14^. During the last ten consecutive days of rat model building, daily vaginal smears were consistently taken from each rat between 9:00 and 10:00 a.m. to identify their estrous cycles.

### Establishment of M617-treated rat model

Rats were randomly divided into four groups namely: control, darkness, DL.reuteri, and DL+M617 groups (*n* = 6/group). The control group was kept under in a 12:12 h light-dark cycle, receiving daily oral gavage of saline for 8 weeks and daily intraperitoneal injection of saline during the last 4 weeks. The darkness group and DL.reuteri group were daily fed on saline or *L. reuteri* (10^10^ CFU/mL, 100 mg/d) respectively by oral gavage during 8-week dark housing. Meanwhile, they received daily intraperitoneal injection of saline during the last 4 weeks. The DL+M617 group was orally treated with *L. reuteri* (10^10^ CFU/mL, 100 mg/d) once a day during 8-week dark housing and was intraperitoneally injected with M617 which is a selective GALR1 agonist (10 nmoL/d/kg BW) (#2697, Tocris Bioscience, Bristol, UK) during the last 4 weeks^59^.

### Establishment of M40-treated rat model

Rats were randomly divided into three groups namely: control, darkness, and darkness+M40 groups (*n* = 8/group). The control group was kept under a circadian rhythm of 12:12 h light-dark cycle and daily intraperitoneally injected with saline during the last 4 weeks. The darkness group and darkness+M40 group were kept in constant darkness for 8 weeks and received daily intraperitoneal injections of saline or M40 which is a non-selective galanin receptor antagonist (30 nmoL/d/kg BW) (#3425, Tocris Bioscience) respectively during the last 4 weeks^60^.

### Establishment of capric acid-treated rat model

Rats were randomly divided into four groups namely: control, darkness, DL.reuteri, and DL+capric acid groups (*n* = 6/group). The control group was kept under in a 12:12 h light-dark cycle, receiving daily oral gavage of saline for 8 weeks and daily subcutaneous injection of corn oil during the last 4 weeks. The darkness group and DL.reuteri group were daily fed on saline or *L. reuteri* (10^10^ CFU/mL, 100 mg/d) respectively by oral gavage during 8-week dark housing, and they received daily subcutaneous injection of corn oil during the last 4 weeks. The DL+capric acid group was orally treated with *L. reuteri* (10^10^ CFU/mL, 100 mg/d) once a day during 8-week dark housing and was subcutaneously injected with capric acid (250 mg/d/kg BW) (#A502170, Sangon Biotech, Shanghai, China) during the last 4 weeks^51,61^.

### Cell culture

The human cell line HepG2 was obtained from a Cell Resource Center (Shanghai Institutes for Biological Sciences, Chinese Academy of Sciences, Shanghai, China). HepG2 cells were cultured in high glucose-DMEM/F12 (#11965-092, Gibco, NY, USA) containing 10% fetal bovine serum (FBS) (#04-001-1ACS, Biological Industries, Israel) at 37 °C in a humidified atmosphere, with 5% CO_2_. The cell lines were routinely subcultured every 2 or 3 days.

### Small interfering (si) RNA transfection with liposome

Transfections of siRNA (GenePharma, Shanghai, China) were performed using Lipofectamine 3000 (#L3000-015, Invitrogen, Carlsbad, CA, USA) and Opti-MEM (#31985070, Gibco) according to the manufacturer’s instructions. HepG2 cells were further incubated for 48 h before detecting the efficiency of knockdown and the abundance of target genes. The specific sequences of target genes are as follows:

*GALR1* siRNA, 5′-GCAAGUUCAUCCACUACUUTT-3′,

*GALR2* siRNA, 5′-GCAAGGUGACACGCAUGAUTT-3′,

*NR1D1* siRNA, 5′-CCCACCUACUUCCCACCAUTT-3′,

*NR1D2* siRNA, 5′-GCAUGGUUCUACUGUGUAATT-3′,

non-specific scrambled siRNA, 5′-UUCUCCGAACGUGUCACGUTT-3′.

### Plasmids transfection with liposome

Lipofectamine 3000 and P3000 (Invitrogen) were mixed with pTSB02-GFP-PURO-GALR1 (Transheep, Shanghai, China), or pTSB02-GFP-PURO-GALR2 (Transheep), or pTSB02-GFP-PURO-NR1D1 (Transheep), or pTSB02-GFP-PURO-NR1D2 (Transheep) in Opti-MEM and then added into cells according to the manufacturer’s instructions. HepG2 cells were further incubated for 72 h before detecting the efficiency of overexpression and the abundance of target genes.

### Real-time quantitative polymerase chain reaction (RT-qPCR)

Total RNA from HepG2 cells, liver, and ileum tissues from rat models was extracted using animal total RNA isolation kit (#RE-03014, FOREGENE, Chengdu, China) and then reversely transcripted into cDNA (#RR037A, Takara Bio, Dalian, China). The mRNA expression of target genes was detected using RT-qPCR (Applied Biosystems, CA, USA). Then the results were analyzed by ΔΔCt method. The target gene/*β-ACTIN* ratio was taken to be our target mRNA level. The primer sequences used for targeting genes are presented in Supplementary Table 1.

### Western blot analysis

Forty μg protein was loaded onto 10% SDS gel and then the protein was transferred to nitrocellulose membrane (Merck Millipore, Massachusetts, USA). The nonspecific binding sites of membrane were blocked and incubated with diluted GALR1 antibody (1:500; #47567, Signalway Antibody, Maryland, USA), GALR2 antibody (1:500; #26459-1-AP, Proteintech, Wuhan, China), NR1D1 antibody (1:1000, #13418 S, Cell Signaling Technology, Massachusetts, USA; 1:1000, #14506-1-AP, Proteintech), NR1D2 antibody (1:500; #13906-1-AP, Proteintech), SREBP1 antibody (1:1000; #41878, Signalway Antibody), LXRa antibody (1:1000; #ab176323, Abcam, Cambridge, UK), RXRa antibody (1:1000; #ab125001, Abcam), INSIG2 antibody (1:500; #24766-1, Proteintech), P-AKT antibody (1:1000; #4060, Cell Signaling Technology), T-AKT antibody (1:1000; #4691, Cell Signaling Technology), P-ERK antibody (1:1000; #4370, Cell Signaling Technology) and T-ERK antibody (1:1000; #4695, Cell Signaling Technology) at 4°C for overnight. Then the nitrocellulose membrane was incubated with diluted matched peroxidase-conjugated secondary antibodies (1:5000; Signalway Antibody) for 1 h at room temperature. At last, the protein signals were detected with Electrochemiluminescence Western Blotting Substrate ((Merck Millipore) using a G-Box iChemi Chemiluminescence image capture system (Syngene, Cambridge, UK). The ratio of target protein to GAPDH (1:5000; #60004-1-Ig, Proteintech) was detected using software. Original blots were provided in the Supplementary Information. All blots were processed in parallel and derive from the same experiment.

### Hematoxylin and eosin staining

Rat ovary, adipose, and ileum tissues were collected and fixed with 4% paraformaldehyde and embedded in paraffin. Five μm-thick tissue sections were prepared which followed by deparaffinization and rehydration through a graded ethanol series. Then the tissue sections were stained in hematoxylin for 5 min and differentiated by hydrochloric acid for 30 s. Finally, the sections were incubated in eosin for 2 min before coving the slide and visualization using a microscope (Zeiss, Oberkochen, Germany).

### Intestine permeability detection

Rats were fasted but allowed water for 6 h. FITC-dextran (#FD4, Sigma-Aldrich, St. Louis, USA) was then given orally (50 mg/100 g BW). At 2 h after gavage, blood was collected and the fluorescence of serum samples was measured (excitation: 490 nm; emission: 520 nm)^62^. Fluorescence intensity of each sample was normalized to the control group.

### Transmission electron microscope

Fresh rat liver tissue (1 mm^3^ in size) was washed with phosphate buffer saline (PBS) and then fast fixed with 2.5% glutaraldehyde in phosphate buffer at room temperature for 1 h and then at 4°C overnight. After fixation, the samples were rinsed three times in PBS before postfixing with 1% osmium tetroxide for 2 h at 4°C. After dehydration in ethanol, the samples were infiltrated and embedded. This was followed by sectioning to ~70 nm. The sample sections were then mounted on copper grids, stained with lead citrate, and observed with an electron microscope (Hitachi HT7800, Tokyo, Japan).

### Oil red O staining

HepG2 cells were plated onto six-well plates and cultured until they were 70 % confluent. After starvation with 1% bovine serum albumin (BSA) for 24 h, cells were treated with oleic acid (#A502071, Sangon Biotech) and palmitic acid (#A600497, Sangon Biotech) at a ratio of 4:1 (OPA) at 1 mM for 18 h. Then HepG2 cells were washed with PBS, and fixed with 4% paraformaldehyde for 30 min at room temperature. The cells were then washed three times with PBS and incubated with 60% isopropanol for 5 min, followed by staining with 0.5 mL of 60% Oil Red O staining solution (#O0625, Sigma-Aldrich) for 1 h. After additional washing with PBS, images were captured under a microscope (Zeiss).

Frozen rat liver was prepared for 5 μm-thick tissue sections. After restoring to normal temperature, the sections were fixed with 4% paraformaldehyde and stained with 60% Oil Red O staining solution. After washing with PBS, the sections were incubated with 75% ethanol, followed by staining with hematoxylin for 5 min before coving the slide and visualization using a microscope (Zeiss).

### Nile red staining

HepG2 cells were treated with BSA and OPA as described above. Washed with PBS and fixed with 4% paraformaldehyde, the cells were then stained with 1 ug/mL Nile Red staining solution (#19123, Sigma-Aldrich) in darkness for 5 min at 37 °C. After additional washing with PBS, the nuclei were counterstained with 4′, 6′-diamidino-2-phenylindole (blue) in darkness for 20 min at room temperature. Images were captured under a microscope (Zeiss) and analyzed with Image J software.

### ELISA

The concentrations of galanin, LH, FSH, testosterone, SHBG, cortisol, and corticosterone in rat sera were detected using Rat Galanin ELISA Kit (#MBS2512765, Mybiosource, San Diego, USA), Rat LH ELISA Kit (#MBS2018978, Mybiosource), Rat FSH ELISA Kit (#EKU04249, Biomatik, Ontario, Canada), Testosterone ELISA Kit (#582701, Cayman Chemical, Michigan), Rat SHBG ELISA Kit (#MBS261678, Mybiosource), Cortisol Parameter Assay Kit (#KGE008B, R&D systems, Minnesota, USA), and Corticosterone Parameter Assay Kit (#KGE009, R&D), respectively. The concentrations of TG, total CHOL, HDL-C, LDL-C, and NEFA in rat sera and liver tissues were detected using Triglyceride Quantification Assay Kit (#ab65336, Abcam), HDL and LDL/VLDL Cholesterol Assay Kit (#ab65390, Abcam), Rat LDL-C ELISA Kit (#CSB-E16561r, Cusabio, Wuhan, China), and Free Fatty Acid Quantification Assay Kit (#ab65341, Abcam), respectively. We carried out all procedures following the recommended instructions provided by kit’s manufacturers.

### Glucose tolerance test

Rats were fasted for 16 h (5 p.m. to 9 a.m.) with free access to water and then intraperitoneally injected with D-glucose (2 g/kg BW) in an alternating order. The blood glucose level was measured in tail vein blood before and 30, 60, 90, and 120 min after D-glucose injection with an Accu-Chek glucose monitor (Roche, Basel, Switzerland). The area under the curve (AUC) was calculated to evaluate the glucose tolerance.

### RNA sequencing

Total RNA was extracted from 24 rat liver samples using TransZol Up Plus RNA Kit (#ER501-01, TransGen Biotech, Beijing, China) and checked for an RNA integrity number (RIN) using Agilent Bioanalyzer 2100 (Agilent technologies, CA, USA). Qualified total RNA (RIN ≥ 7) was further purified by RNAClean XP Kit (#A63987, Beckman Coulter, CA, USA) and RNase-Fr DNase Set (#79254, Qiagen, Hilden, Germany). The libraries were prepared with VAHTS Universal V6 RNA-seq Library Prep Kit for Illumina® (#NR604-02, Vazyme Biotech, Nanjing, China) and sequenced using the Illumina HiSeq 2500 platform (San Diego, CA, USA). The poor-quality reads, the adapters, and the polluted reads of the raw reads were trimmed using Seqtk software. Then the genome mapping of clean reads was identified using Hisat2 (2.0.4). The fragments per kilobase of exon model per million mapped reads values were calculated with StringTie (1.3.0) based on the consensus transcript, followed by the Ballgown procedure to identify differentially expressed genes with the criteria of |fold change | > 0.6 in the log_2_ ratio value and raw *P* < 0.05.

An unsupervised co-expression network analysis of 2,724 genes (those with low variation in expression, standard deviation ≤ 0.5, were filtered from all 14,544 annotated genes and 23 liver samples) was performed using WGCNA (R package WGCNA). The scale-free topology overlap matrix was computed using a best soft threshold power of 20 obtained from WGCNA function “pickSoftThreshold”. For each identified module of co-expression biomolecules, Spearman’s correlations between module eigengenes and phenotype data were calculated (|ρ | > 0.3, *P* < 0.05). GO and KEGG enrichment analyses were performed using the Database for Annotation, Visualization, and Integrated Discovery (DAVID, 6.8) (https://david.ncifcrf.gov/) and visualized using ggplot2 package. GSEA with GO biological processes and KEGG pathways^63^ were conducted on the hepatic gene expression profiling data of control and darkness rats. Statistical significance was set at nominal *P* < 0.05.

### Faecal DNA extraction and 16 S rRNA gene sequencing

Faecal samples for 16 S rRNA gene sequencing were collected after 8-week treatment from *L. reuteri*-treated rat model and M40-treated rat model. Total genomic DNA was extracted from faecal samples using QIAamp Fast DNA Stool Mini Kit (#51604, Qiagen) according to the manufacturer’s protocol. Bacterial primers 338 F (5′-ACTCCTACGGGAGGCAGCAG-3′) and 806 R (5′-GGACTACHVGGGTWTCTAAT-3′) targeting the V3-V4 hyper-variable region of 16 S rRNA gene was used for PCR amplification. PCR amplicons were sequenced using the MiSeq Illumina platform. Raw sequence reads were demultiplexed, quality filtered, clustered into Operational Taxonomic Units (OTUs), and aligned to the Silva bacterial 16 S rRNA gene dataset (release 123) and Greengenes database with 97% sequence similarity in Quantitative Insights Into Microbial Ecology (QIIME, 1.9.1). Prior to downstream analysis, we removed the singleton OTUs and rarified all samples to the lowest depth of sequencing. α-Diversity was calculated using Chao, observed species, coverage, and Shannon metrics. β-Diversities were calculated using unweighted Unifrac and shown in principal coordinate analysis (PCoA) plots. To compare the relative abundance of bacterial taxa between groups, metastats analysis was applied. Heatmap was generated based on normalized bacteria abundance using a R function heatmap. LEfSe method was performed to discover the high-dimensional biomarker that characterizes the microbial differences between groups. Predictive function analysis was performed with PICRUSt2.0 (Phylogenetic Investigation of Communities by Reconstruction of Unobserved States) based on the KEGG Orthology (KO) classification.

### Untargeted metabolomics

Serum samples and faecal samples for untargeted metabolomics were collected after 8-week treatment from *L. reuteri*-treated rat model. Aliquots of 100 μL were drawn from each serum sample to which 300 μL methanol was added. After thorough vortex and centrifugation, an aliquot of 200 μL supernatant was added to 5 μL internal standard (140 μg/mL, DL-o-Chlorophenylalanine) and then transferred to a vial for liquid chromatograph mass spectrometer (LC-MS) analysis. Nexera ultra-high performance liquid chromatograph (UHPLC) (LC-30A) (Shimadzu, Japan) was coupled to a Triple TOF 6600 plus (AB Sciex, CA, USA) mass spectrometer equipped with an electrospray ionization (ESI) source. After centrifugation, 4 μL supernatant was injected. During LC separation using an Acquity UPLC HSS T3 column (100 mm × 2.1 mm × 1.8 μm, Waters, USA), the column temperature was set at 40 °C with an elution rate of 0.3 mL/min. The mobile phases consisted of 0.1% formic acid in water (A) and 0.1% formic acid in acetonitrile (B). The LC eluents were run following the gradient: 5% B, 0–2 min; 5%–70% B, 2–5 min; 70%–90% B, 5–14 min; 90%–100% B, 14–16 min; 100% B, 16–22 min; 100%–5% B, 22–22.1 min; 5% B, 22.1–27 min. The HESI ion source temperature was set at 550 °C, and ion spray voltage was set at 5.5 kV and −4.5 kV for positive and negative ion modes, respectively.

For each faecal sample, 50 mg feces was aliquoted and extracted by 800 μL ice-cold methanol: ddH_2_O (4:1, v/v). After vortex and centrifugation, an aliquot of 200 μL supernatant was added to 5 μL internal standard and transferred for LC-MS analysis. Extracts of equivalence to 0.625 mg raw feces (10 μL supernatant) was injected and subject to a chromatographic separation. Mobile phases were comprised of 0.05% formic acid either in water (A) or in acetonitrile (B). The column temperature, elution rate, elution gradient, and HESI ion source parameters remained as described above.

Quality control (QC) procedures were performed including timely mass calibration, sample randomization, and intermittent QC injection. Raw data were imported to MS-DIAL software for peak picking, peak alignment, gap filling, and sample normalization. After data management, a total of 166 and 170 metabolites were identified out of 10,323 (ESI+) and 8,788 (ESI-) ion features in faecal samples, and a total of 138 and 104 metabolites were recognized out of 9,713 (ESI+) and 7,156 (ESI-) ion features in serum samples. The peak area of each chromatographic peak in the full-scan MS represented the relative content of corresponding metabolites. The annotation for known metabolites was based on the mass charge ratio (m/z) and accurate mass against the online METLIN Metabolomics Database. Predictive pathway analysis was performed with MetaboAnalyst 5.0 and visualized with ggplot2 package.

### Statistical analysis

Results are presented as mean ± SEM. Statistical analysis was performed using IBM SPSS Statistics and Prism 8 (GraphPad Software Inc.). If not specified in the figure legends, the data were initially subjected to Kolmogorov-Smirnov tests to assess deviation from Gaussian distribution. The unpaired Student’s t-test or one-way analysis of variance (ANOVA) was used where data was normally distributed data. These analyses were followed by the Newman–Keuls multiple comparison test. For skewed data, we applied Kruskal–Wallis test followed by Dunn’s multiple comparison test. The correlation of two datasets was analyzed using Spearman’s rank correlation coefficient. For all tests, a two-tailed *P* < 0.05 was considered as statistically significant.

SIMCA-P 14.0 software (Umetrics AB, Umea, Sweden) was used to perform multivariate statistical analysis screening for ion features of distinct group differences, including PCA, PLS-DA, and OPLS-DA. Variable importance in the projection (VIP) value (threshold >1) of the OPLS-DA model combined with t-test (*P* < 0.05) of the peak area was applied to figure out the differentially expressed metabolites between two groups.

### Reporting summary

Further information on research design is available in the Nature Research Reporting Summary linked to this article.

## Supplementary information

Reporting summary

Supplementary Information

Supplementary Data 1

Supplementary Data 2

Supplementary Data 3

Supplementary Data 4

Supplementary Data 5

Supplementary Data 6

Supplementary Data 7

Supplementary Data 8

## Data Availability

All sequencing data have been deposited to the Sequence Read Archive of the National Center for Biotechnology Information. The 16 S rRNA gene sequencing data are available under the accession number PRJNA716107 (*L. reuteri*-treated rat model) and PRJNA964489 (M40-treated rat model). Hepatic mRNA sequencing data is available with Gene Expression Omnibus: GSE169501. Other data generated or analyzed during this study are included in this article and its supplementary files and from the Lead Contact.
