# Supplementary material for: Alleviation of Limosilactobacillus reuteri in polycystic ovary syndrome protects against circadian dysrhythmia-induced dyslipidemia via capric acid and GALR1 signaling
Source: NPJ Biofilms Microbiomes. 2023 Jul 8;9:47. doi: 10.1038/s41522-023-00415-2 (PMC10329655; doi:10.1038/s41522-023-00415-2)
Supplement: Supplementary file 1 — Reporting summary [file 41522_2023_415_MOESM1_ESM.pdf]

nature portfolio

Corresponding author(s): Zi-Jiang Chen and Yanzhi Du

Last updated by author(s): May 2, 2023

Reporting Summary

Nature Portfolio wishes to improve the reproducibility of the work that we publish. This form provides structure for consistency and transparency in reporting. For further information on Nature Portfolio policies, see our Editorial Policies and the Editorial Policy Checklist.

Statistics

For all statistical analyses, confirm that the following items are present in the figure legend, table legend, main text, or Methods section.

n/a	Confirmed	
☐	☒	The exact sample size (*n*) for each experimental group/condition, given as a discrete number and unit of measurement
☐	☒	A statement on whether measurements were taken from distinct samples or whether the same sample was measured repeatedly
☐	☒	The statistical test(s) used AND whether they are one- or two-sided<br>*Only common tests should be described solely by name; describe more complex techniques in the Methods section.*
☒	☐	A description of all covariates tested
☐	☒	A description of any assumptions or corrections, such as tests of normality and adjustment for multiple comparisons
☐	☒	A full description of the statistical parameters including central tendency (e.g. means) or other basic estimates (e.g. regression coefficient) AND variation (e.g. standard deviation) or associated estimates of uncertainty (e.g. confidence intervals)
☒	☐	For null hypothesis testing, the test statistic (e.g. *F*, *t*, *r*) with confidence intervals, effect sizes, degrees of freedom and *P* value noted<br>*Give P values as exact values whenever suitable.*
☒	☐	For Bayesian analysis, information on the choice of priors and Markov chain Monte Carlo settings
☒	☐	For hierarchical and complex designs, identification of the appropriate level for tests and full reporting of outcomes
☒	☐	Estimates of effect sizes (e.g. Cohen's *d*, Pearson's *r*), indicating how they were calculated

Our web collection on statistics for biologists contains articles on many of the points above.

Software and code

Policy information about availability of computer code

Data collection	The RT-qPCR results were collected using Applied Biosystems ViiA7. The protein signals of western blot analysis were collected using Syngene software. The microscope results were visualized using Zeiss software. Other data were collected with Microsoft Excel (2013).
Data analysis	GraphPad Prism (8.0) and SPSS (23.0) were used for statistical treatment. Nile Red staining was analyzed with Image J software (1.8.0). The raw reads of RNA sequencing were trimmed using Seqtk. Then the genome mapping of clean reads was identified using Hisat2 (2.0.4) and Ensembl for Rnor (6.0). The FPKM values were calculated with StringTie (1.3.0) based on the consensus transcript. GO and KEGG enrichment analyses were performed using DAVID (6.8) (https://david.ncifcrf.gov/) and visualized using ggplot2 package. Raw sequence reads of 16S rRNA sequencing were demultiplexed, quality filtered, clustered into OTUs, and aligned to the Silva bacterial 16S rRNA gene dataset (release 123) and Greengenes database with 97% sequence similarity in QIIME (1.9.1). Predictive function analysis was performed with PICRUSt2.0. SIMCA-P 14.0 software was used to perform multivariate statistical analysis for metabolites. Predictive pathway analysis of metabolites was performed with MetaboAnalyst (5.0) and visualized with ggplot2 package.

For manuscripts utilizing custom algorithms or software that are central to the research but not yet described in published literature, software must be made available to editors and reviewers. We strongly encourage code deposition in a community repository (e.g. GitHub). See the Nature Portfolio guidelines for submitting code & software for further information.

Data

Policy information about availability of data

All manuscripts must include a data availability statement. This statement should provide the following information, where applicable:

- Accession codes, unique identifiers, or web links for publicly available datasets
- A description of any restrictions on data availability
- For clinical datasets or third party data, please ensure that the statement adheres to our policy

All sequencing data have been deposited to the Sequence Read Archive of the National Center for Biotechnology Information. The 16S rRNA gene sequencing data are available under the accession number PRJNA716107 (L. reuteri-treated rat model) and PRJNA964489 (M40-treated rat model). Hepatic mRNA sequencing data is available with Gene Expression Omnibus: GSE169501.

Research involving human participants, their data, or biological material

Policy information about studies with human participants or human data. See also policy information about sex, gender (identity/presentation), and sexual orientation and race, ethnicity and racism.

Reporting on sex and gender	N/A
Reporting on race, ethnicity, or other socially relevant groupings	N/A
Population characteristics	N/A
Recruitment	N/A
Ethics oversight	N/A

Note that full information on the approval of the study protocol must also be provided in the manuscript.

Field-specific reporting

Please select the one below that is the best fit for your research. If you are not sure, read the appropriate sections before making your selection.

☒ Life sciences ☐ Behavioural & social sciences ☐ Ecological, evolutionary & environmental sciences

For a reference copy of the document with all sections, see nature.com/documents/nr-reporting-summary-flat.pdf

Life sciences study design

All studies must disclose on these points even when the disclosure is negative.

Sample size	In the animal experiments, each group involved 6 or 8 rats in order to obtain statistical significance. The exact sample sizes were listed in figure legends.
Data exclusions	No data was excluded.
Replication	All data are representative of three or more independent experiments. All attempts at replication were successful.
Randomization	Six-week-old female rats were randomly divided into experimental groups, with 6 or 8 per group.
Blinding	Histological analysis of the rat ovary, adipose, and ileum samples were blindly performed and analyzed. Transmission electron microscope, Oil Red O staining, and Nile Red staining were blindly performed and analyzed. During the treatment of live animals it was not blinded, but investigators were blinded during sample collection.

Reporting for specific materials, systems and methods

We require information from authors about some types of materials, experimental systems and methods used in many studies. Here, indicate whether each material, system or method listed is relevant to your study. If you are not sure if a list item applies to your research, read the appropriate section before selecting a response.

Materials & experimental systems

n/a	Involved in the study
☐	☒ Antibodies
☒	☐ Eukaryotic cell lines
☒	☐ Palaeontology and archaeology
☐	☒ Animals and other organisms
☒	☐ Clinical data
☒	☐ Dual use research of concern
☒	☐ Plants

Methods

n/a	Involved in the study
☒	☐ ChIP-seq
☒	☐ Flow cytometry
☒	☐ MRI-based neuroimaging

Antibodies

Antibodies used

For western blot analysis, primary antibodies used and dilution ratios are:
GALR1 antibody (1:500; #47567, Signalway Antibody, Maryland, USA)
GALR2 antibody ((1:500; #26459-1-AP, Proteintech, Wuhan, China)
NR1D1 antibody (1:1000; #13418S, Cell Signaling Technology, Massachusetts, USA)
NR1D2 antibody (1:500; #13906-1-AP, Proteintech, Wuhan, China)
SREBP1 antibody (1:1000; #41878, Signalway Antibody, Maryland, USA)
LXRa antibody (1:1000; #ab176323, Abcam, Cambridge, UK)
RXRa antibody (1:1000; #ab125001, Abcam, Cambridge, UK)
INSIG2 (1:500; #24766-1, Proteintech, Wuhan, China)
P-AKT antibody (1:1000; #4060, Cell Signaling Technology, Massachusetts, USA)
T-AKT antibody (1:1000; #4691, Cell Signaling Technology, Massachusetts, USA)
P-ERK antibody (1:1000; #4370, Cell Signaling Technology, Massachusetts, USA)
T-ERK antibody (1:1000; #4695, Cell Signaling Technology, Massachusetts, USA)
GAPDH antibody (1:5000; #60004-1-Ig, Proteintech, Wuhan, China)

Validation

The antibodies used in our study were validated by the manufacturers and used according to the manufacturers' instructions.
GALR1
Applications: WB
Species Reactivity: Human, Mouse, Rat
https://www.sabbiotech.com.cn/g-150433-GALR1-Antibody-47567.html

GALR2
Applications: WB, IF
Species Reactivity: Human, Mouse
https://www.ptgcn.com/products/GALR2-Antibody-26459-1-AP.htm#product-information

NR1D1
Applications: WB, IP, ChIP
Species Reactivity: Human, Mouse, Rat
https://www.cellsignal.com/products/primary-antibodies/rev-erba-e1y6d-rabbit-mab/13418?site-search-type=Products&N=4294956287&Ntt=%2313418&fromPage=plp&_requestid=3024533

NR1D2
Applications: WB, ELISA
Species Reactivity: Human, Mouse
https://www.ptgcn.com/products/NR1D2-Antibody-13906-1-AP.htm

SREBP1
Applications: WB, ELISA
Species Reactivity: Human, Mouse, Rat
https://www.sabbiotech.com.cn/g-15561-SREBP-1-Polyclonal-Antibody-41878.html

LXRa
Applications: Flow Cyt, WB, ICC/IF
Species Reactivity: Human (Predicted: Mouse, Rat)
https://www.abcam.cn/lxr-alpha-antibody-epr6508n-ab176323.html

RXRa
Applications: WB, IP, ICC/IF
Species Reactivity:Human, Mouse, Rat
https://www.abcam.cn/retinoid-x-receptor-alpharxra-antibody-epr7106-ab125001.html

INSIG2
Applications: IF, IHC, IP, WB, ELISA
Species Reactivity:Human, Mouse, Rat
https://www.ptgcn.com/products/INSIG2-Antibody-24766-1-AP.htm

P-AKT

Applications: WB, IP, IHC, IF, Flow Cyt
Species Reactivity:Human, Mouse, Rat, Hamster, Monkey, Drosophila melanogaster, Zerafish, Bovine
https://www.cellsignal.cn/products/primary-antibodies/phospho-akt-ser473-d9e-xp-rabbit-mab/4060?site-search-type=Products&N=4294956287&Ntt=p-akt&fromPage=plp

T-AKT
Applications: WB, IP, IHC, IF, Flow Cyt
Species Reactivity:Human, Mouse, Rat, Monkey, Drosophila melanogaster
https://www.cellsignal.cn/products/primary-antibodies/akt-pan-c67e7-rabbit-mab/4691?site-search-type=Products&N=4294956287&Ntt=akt+4691&fromPage=plp&_requestid=6184385

P-ERK
Applications: WB, IP, IHC, IF, Flow Cyt
Species Reactivity:Human, Mouse, Rat, Hamster, Monkey, Drosophila melanogaster, Zerafish, Bovine......
https://www.cellsignal.cn/products/primary-antibodies/phospho-p44-42-mapk-erk1-2-thr202-tyr204-d13-14-4e-xp-rabbit-mab/4370?site-search-type=Products&N=4294956287&Ntt=p-erk+4370&fromPage=plp&_requestid=6184529

T-ERK
Applications: WB, IP, IHC, IF, Flow Cyt
Species Reactivity:Human, Mouse, Rat, Hamster, Monkey, Drosophila melanogaster, Zerafish, Bovine......
https://www.cellsignal.cn/products/primary-antibodies/p44-42-mapk-erk1-2-137f5-rabbit-mab/4695?site-search-type=Products&N=4294956287&Ntt=erk+4695&fromPage=plp

GAPDH
Applications: FC, IF, IP, WB, ELISA
Species Reactivity:Human, Mouse, Rat, Yeast, Plant, Bovine, Branchiostoma belcheri, Caenorhabditis elegans, Canine, Chicken, Cynomorium songaricum, D. pulex, Deer, Duck, Frog
https://www.ptgcn.com/products/GAPDH-Antibody-60004-1-Ig.htm

Animals and other research organisms

Policy information about studies involving animals; ARRIVE guidelines recommended for reporting animal research, and Sex and Gender in Research

Laboratory animals	Six-week-old female Sprague–Dawley Rattus norvegicus (Vital River Laboratory Animal Technology, Beijing, China) were housed in an animal lab under specific pathogen free conditions with three or four rats per cage. All rats were kept at a temperature of 21°C ± 2°C and a humidity of 65% ± 5 %. They received normal chow diet and autoclaved, sterilized water they could freely access ad libitum.
Wild animals	This study did not involve wild animals.
Reporting on sex	Findings apply to only female.
Field-collected samples	This study did not involve field-collected samples.
Ethics oversight	All experimental protocols were reviewed and approved by the Committee on Laboratory Animal Research of Shanghai Jiao Tong University, China.

Note that full information on the approval of the study protocol must also be provided in the manuscript.
