# Supplementary material for: Alleviation of Limosilactobacillus reuteri in polycystic ovary syndrome protects against circadian dysrhythmia-induced dyslipidemia via capric acid and GALR1 signaling
Source: NPJ Biofilms Microbiomes. 2023 Jul 8;9:47. doi: 10.1038/s41522-023-00415-2 (PMC10329655; doi:10.1038/s41522-023-00415-2)
Supplement: Supplementary file 2 — Supplementary Information [file 41522_2023_415_MOESM2_ESM.pdf]

Supplementary Information

Alleviation of *Limosilactobacillus reuteri* in polycystic ovary syndrome protects against circadian dysrhythmia-induced dyslipidemia via capric acid and GALR1 signaling

Shang Li#, Junyu Zhai#, Weiwei Chu, Xueying Geng, Dongshuang Wang, Luwei Jiao, Gang Lu, Wai-Yee Chan, Kang Sun, Yun Sun, Zi-Jiang Chen*, Yanzhi Du*

#These two authors contributed equally to this work

*Corresponding authors:

Zi-Jiang Chen

E-mail: chenzijiang@hotmail.com

Yanzhi Du

E-mail: duyz@sjtu.edu.cn

Supplementary Figure 1

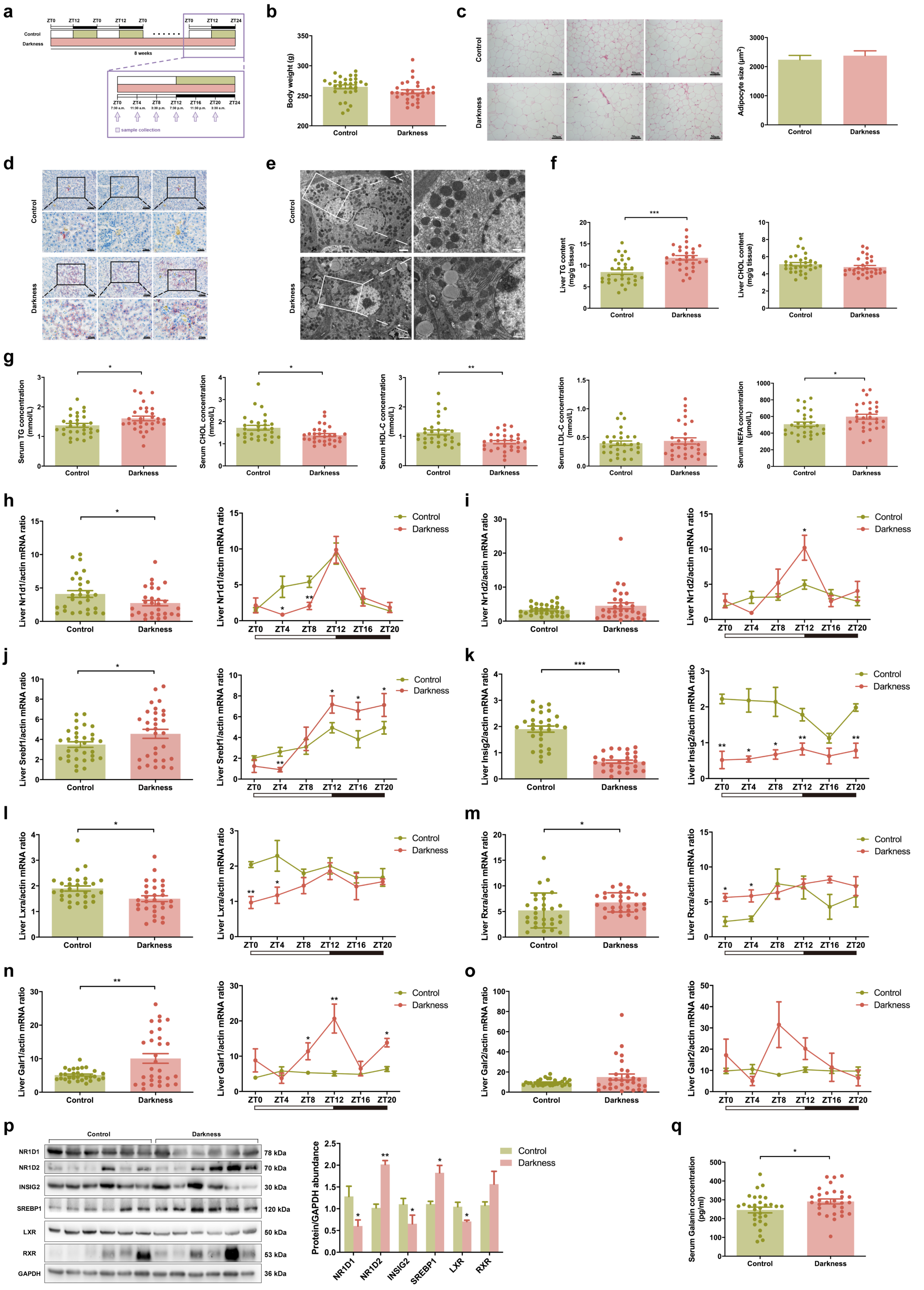

Supplementary Figure 1. Rhythmically aberrant expression of target genes in the liver of control and darkness rats.

(a) Timeline depicting the treatments of darkness exposure and sample collection. Sixty 6-week-old female Sprague-Dawley were equally and randomly divided into 2 groups: control and darkness groups (n = 30 per group). After 8-week housing, 5 rats from each group were killed every 4 hours from ZT0 to ZT20. ZT0 was defined as 7:30 AM according to the lights-on time in the animal lab (n = 5/ZT). **(b)** Body weights of control and darkness rats. **(c)** Representative hematoxylin and eosin staining of adipose. Left, representative images were shown. Right, intensity was quantified. Scale bar: 50 μm. **(d)** Representative hepatic specimens stained with Oil Red O. Scale bar: 50 μm and 25 μm. **(e)** Representative images of liver ultrastructure detected by transmission electron microscope. Scale bar: 40 μm and 1 μm. **(f)** Hepatic contents of TG and CHOL detected by ELISA. **(g)** Serum concentrations of TG, CHOL, HDL-C, LDL-C, and NEFA detected by ELISA. **(h-o)** mRNA abundance of *Nr1d1* **(h)**, *Nr1d2* **(i)**, *Srebf1* **(j)**, *Insig2* **(k)**, *Lxra* **(l)**, *Rxra* **(m)**, *Galr1* **(n)**, and *Galr2* **(o)** in rat liver (n = 5/ZT). **(p)** Protein abundance of NR1D1, NR1D2, SREBP1, INSIG2, LXR, and RXR in rat liver. Left, representative images of western blot were shown. Right, immunoreactive bands were densitometrically quantified. **(q)** Serum galanin concentration detected by ELISA. Statistical analysis was performed with unpaired Student's t-test. Data present means ± SEM. *$P < 0.05$, **$P < 0.01$, ***$P < 0.001$. ZT, zeitgeber time; TG, triglyceride; CHOL, cholesterol; ELISA, enzyme-linked immunosorbent assay; HDL-C, higher high-density lipoprotein-cholesterol; LDL-C, low-density lipoprotein-cholesterol; NEFA, non-esterified fatty acid; NR1D1/2, nuclear receptors subfamily 1, group D, member 1/ 2; SREBF1, sterol regulatory element binding transcription factor 1; INSIG2, insulin induced gene 2; LXRa, liver X receptor a; RXRa, retinoid X receptor a; GALR1/2, galanin receptor 1/2.

Supplementary Figure 2

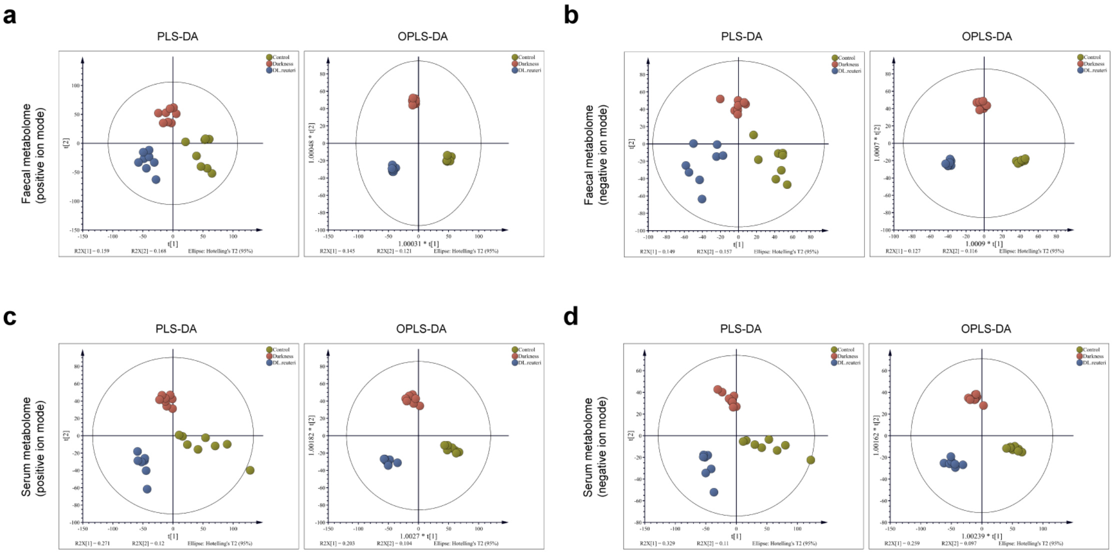

Supplementary Figure 2. Principal component analyses of faecal metabolome and serum metabolome in *L. reuteri*-treated darkness rats.

(a, b) Scatter plot of scores from PLS-DA and OPLS-DA of faecal metabolome in positive ion mode **(a)** and negative ion mode **(b)**. **(c, d)** Scatter plot of scores from PLS-DA and OPLS-DA of serum metabolome in positive ion mode **(c)** and negative ion mode **(d)**. n = 8 per group. *L. reuteri*, *Limosilactobacillus reuteri*; DL.reuteri, darkness+*L. reuteri* (darkness rats following *L. reuteri* treatment); PLS-DA, partial least squares discrimination analysis; OPLS-DA, orthogonal partial least squares discrimination analysis.

Supplementary Figure 3

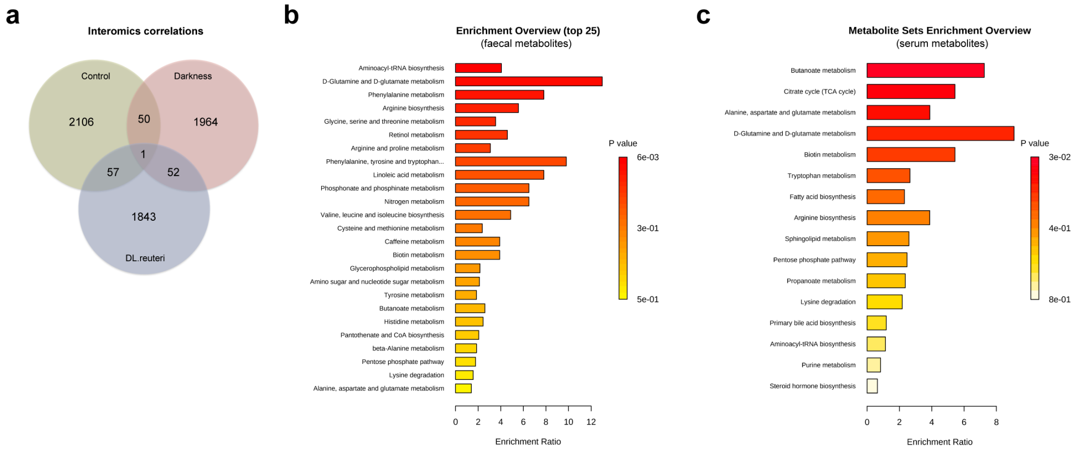

Supplementary Figure 3. Crosstalk among multiomics correlations of faecal microbiome, faecal, and serum metabolomes in *L. reuteri*-treated darkness rats.

(a) Crosstalk among multiomics correlations of all variables for the faecal microbiome, faecal, and serum metabolomes of control, darkness, and DL.reuteri rats. **(b, c)** A total of 57 common correlations were observed in control and DL.reuteri rats but not in darkness rats. The faecal metabolites **(b)** and serum metabolites **(c)** included in the 57 correlations were further used for predictive pathway enrichment. *L. reuteri*, *Limosilactobacillus reuteri*; DL.reuteri, darkness+*L. reuteri* (darkness rats following *L. reuteri* treatment).

Supplementary Figure 4

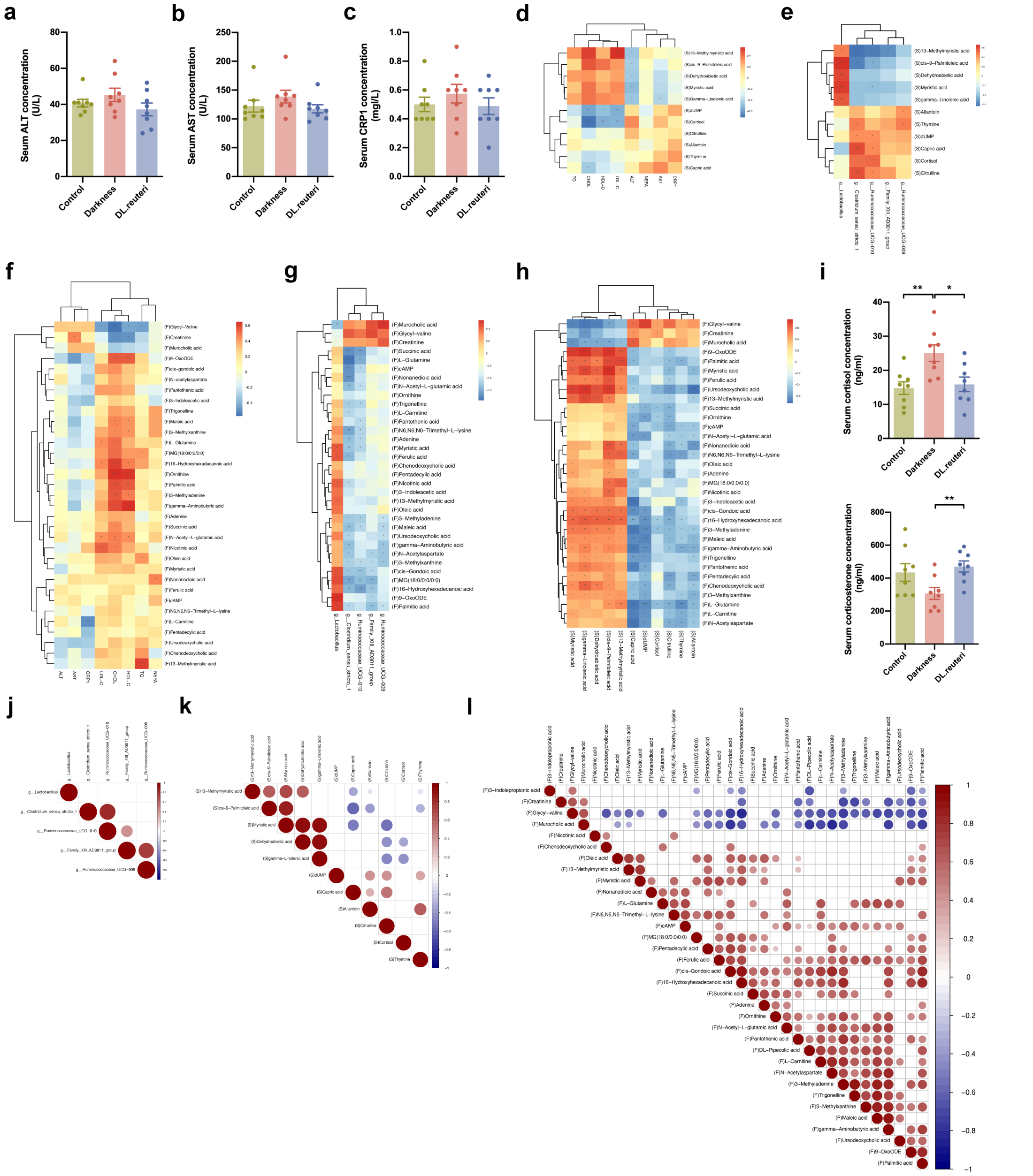

Supplementary Figure 4. Correlation networks of faecal microbiome, faecal, and serum metabolomes in *L. reuteri*-treated darkness rats.

(a-c) Serum concentrations of ALT **(a)**, AST **(b)**, CRP1 **(c)** detected by ELISA. **(d-h)** Spearman rank correlations between differential serum metabolites and clinical biochemical index **(d)**, between differential serum metabolites and genera **(e)**, between differential faecal metabolites and clinical biochemical index **(f)**, between differential faecal metabolites and genera **(g)**, between differential faecal and serum metabolites **(h)**. **(i)** Serum concentrations of cortisol and

corticosterone detected by ELISA. **(j-l)** Spearman rank correlations within differential genera **(j)**, within faecal metabolites **(k)**, and within serum metabolites **(l)** ($P < 0.05$). Statistical analysis (**a-c**, **i**) was performed with one-way ANOVA followed by Newman–Keuls multiple comparison test. n = 8 per group. Data present means ± SEM. *$P < 0.05$, **$P < 0.01$, ***$P < 0.001$. *L. reuteri*, *Limosilactobacillus reuteri*; DL.reuteri, darkness+*L. reuteri* (darkness rats following *L. reuteri* treatment); ALT, alanine aminotransferase; AST, aspartate transaminase; CRP1, c-reactive protein 1; ELISA, enzyme-linked immunosorbent assay.

Supplementary Figure 5

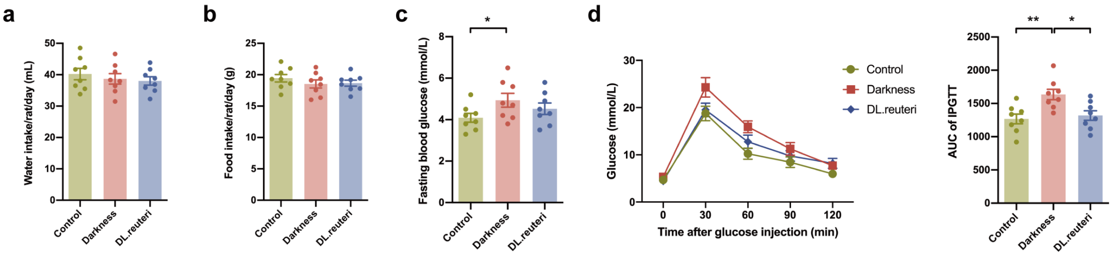

Supplementary Figure 5. Effects of *L. reuteri* on water intake, food intake and glucose metabolism in darkness rats.

(a) Water intake of each group. **(b)** Food intake of each group. **(c)** Fasting blood glucose measured using an Accu-Chek glucose monitor. **(d)** Glucose tolerance test (left) and AUC values (right). Statistical analysis was performed with one-way ANOVA followed by Newman–Keuls multiple comparison test. n = 8 per group. Data present means ± SEM. *$P < 0.05$, **$P < 0.01$. *L. reuteri*, *Limosilactobacillus reuteri*; DL.reuteri, darkness+*L. reuteri* (darkness rats following *L. reuteri* treatment); AUC, area under the curve.

Supplementary Figure 6

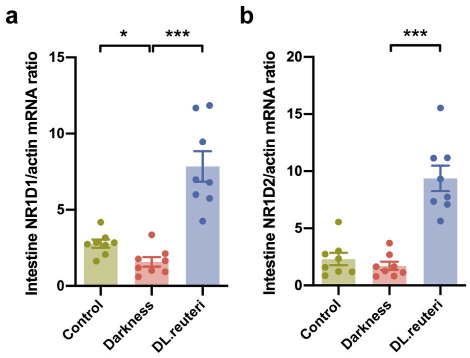

Supplementary Figure 6. Effects of *L. reuteri* on *Nr1d1* and *Nr1d2* expression in the intestine of darkness rats.

mRNA abundance of *Nr1d1* **(a)** and *Nr1d2* **(b)** in the rat ileum. Statistical analysis was performed with one-way ANOVA followed by Newman–Keuls multiple comparison test. n = 8 per group. Data present means ± SEM. **P* < 0.05, ****P* < 0.001. *L. reuteri*, *Limosilactobacillus reuteri*; DL.reuteri, darkness+*L. reuteri* (darkness rats following *L. reuteri* treatment); NR1D1/2, nuclear receptors subfamily 1, group D, member 1/ 2.

Supplementary Figure 7

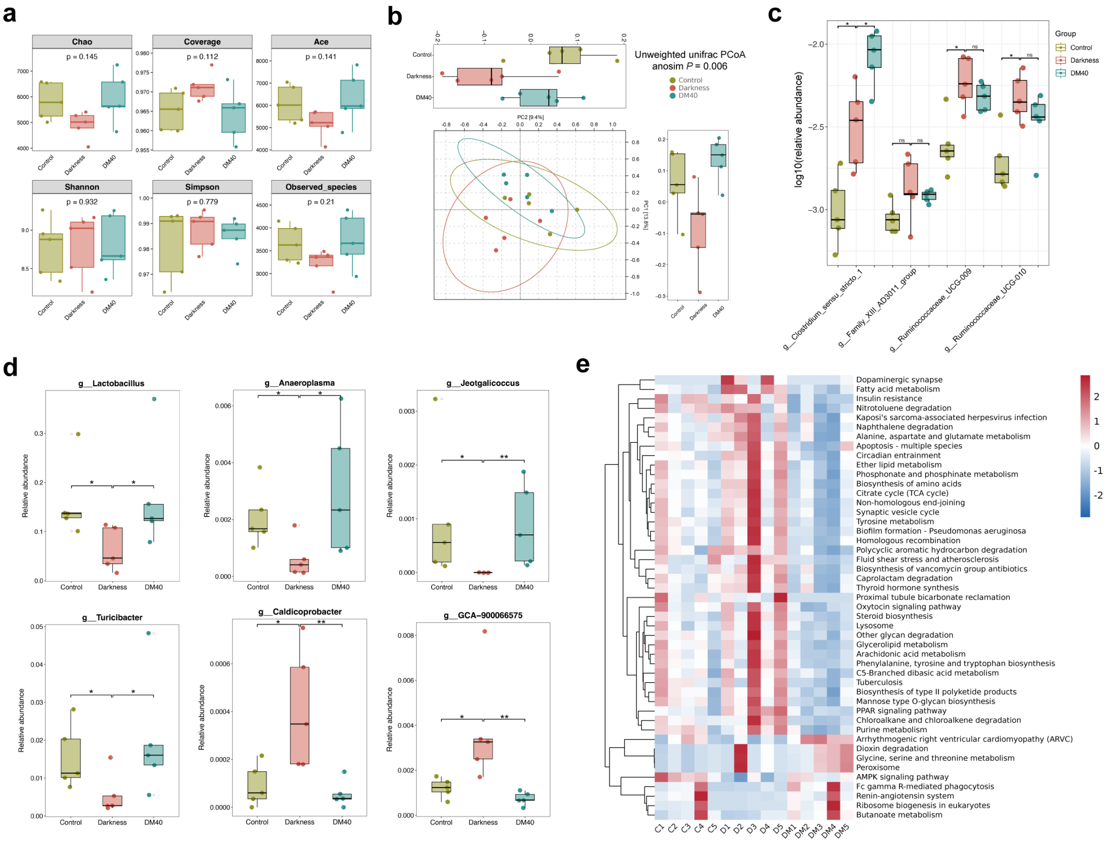

Supplementary Figure 7. Faecal microbiome analysis of M40-treated darkness rats.

(a) Boxplots of a-diversity calculated by Chao, Coverage, Ace, Shannon, Simpson, and Observed species. **(b)** PCoA plot based on unweighted Unifrac distance was used to test the variations of microbial communities among groups. Analysis of similarities (anosim): $P = 0.006$ with 999 permutations. **(c)** Relative abundance of *Clostridium sensu stricto 1*, *Family XIII AD3011 group*, *Ruminococcaceae UCG-009*, and *Ruminococcaceae UCG-010* in control, darkness, and darkness+M40 rats. **(d)** Relative abundance of differential genera (highly or lowly expressed in darkness rats compared with control and darkness+M40 rats, $P < 0.05$). Statistical analysis (**c**, **d**) was performed with Kruskal–Wallis test followed by Dunn's multiple comparison test. **(e)** All differential KEGG pathways (Krusal-Wallis analysis, $P < 0.05$) predicted by PICRUSt2.0 analysis of faecal microbiome. The boxplot elements were defined as following: center line, median; box limits, upper, and lower quartiles; whiskers, 1.5 × interquartile range. Points outside the whiskers represented outlier samples. n = 5 per group. *$P < 0.05$, **$P < 0.01$. DM40, darkness+M40

(darkness rats following M40 treatment); PCoA, principal coordinate analysis; KEGG, Kyoto Encyclopedia of Genes and Genomes.

Supplementary Table 1. Primer sequences of the RT-qPCR tested genes.

Gene	Primer forward (5' to 3')	Primer reverse (5' to 3')
Galr1 (rat)	TGTTCAAGTGCCGTGTTTGC	GAGGGCGGGGTATCTATTCG
Galr2 (rat)	ACCCCTATTTTTCGCGCTCA	TCGTCCAGGGTGTAGATGGT
Galr3 (rat)	ACCTCACCATGTATGCCAGC	CTTAGGTAGGGCGCGGAAAA
Nr1d1 (rat)	ACTTCCCACCATCACCTACTG	GGGGAGCTATCATCACTGAGA
Nr1d2 (rat)	CAGGAGGTGTGATTGCCTACA	GGACGAGGACTGGAAGCTATT
Srebf1 (rat)	CAAGGCCATCGACTACATCCG	CACCACTTCGGGTTTCATG
Lxra (rat)	CTGATTCTGCAACGGAGTTGT	GACGAAGCTCTGTCGGCTC
Rxra (rat)	ATGGACACCAAACATTTCCTGC	CCAGTGGAGAGCCGATTCC
Insig2 (rat)	TAAATCACGCCAGTGCTAAAGT	GGTGACAACGGTTGCTAAGAAAG
Zo1 (rat)	ACCTTGAGCAGCCACCATAC	CGAGTTGGGTAGGGCTGTTT
Ocln (rat)	TGTATGGCGGAGAGATGCAC	GCGATGCACATCACGATGAC
Cldn1 (rat)	TGGGTTTCATCCTGGCTTCG	AGCAGTCACGATGTTGTCCC
Cldn2 (rat)	CCAACTGGTGGGCTACATCC	CCTTGGAAAAGCCAACCGC
Cldn3 (rat)	GAAGTACGCACCCACCAAGA	GTCCTTACGGTCATAGGCGG
Cldn5 (rat)	GGGCGTCCAGAGTTCAGTTT	TCTTCTTGTCGTAATCGCCGT
Cldn7 (rat)	CGAGTTTGGTCCTGCCATCT	TGGACTTAGGGTAGGAGCGG
Akp (rat)	TCATGTTCGACTCCGCCATC	AGTGATGAGCGTTAGCGTGT
β-Actin (rat)	GGCCAACCGTGAAAAGATGACC	AACCCTCATAGATGGGCACAG
GALR1 (human)	ATCTGCTTCTGCTATGCCAAG	CAGTGGGCGGTGATTCTGA
GALR2 (human)	GCCCTACCTGAGCTACTACC	GATGAGGATCATGCGTGTCAC

NR1D1 (human)	ATCGTCCGCATCAATCGCAA	CTGCTTCTCTCGTTTGGGGAT
NR1D2 (human)	TCATGCTTGCGAAGGCTGTAA	CGCTTAGGAATACGACCAAACC
SREBF1 (human)	CGGAACCATCTTGGCAACAGT	CGCTTCTCAATGGCGTTGT
LXRa (human)	ACAAAAGCGGAAAAAGGGGC	AAGAATCCCTTGCAGCCCTC
RXRa (human)	AACATTTCCTGCCGCTCGATTT	AGGGTGCTGATGGGAGAATGC
INSIG2 (human)	TAATGCGGTGTGTAGCAGTCT	GTCCAATGGATAGTGCAGCCA
β-ACTIN (human)	GGGAAATCGTGCGTGACATTAAG	TGTGTTGGCGTACAGGTCTTTG

Galr, *galanin receptor*; *Nr1d1/2*, *nuclear receptors subfamily 1, group D, member 1/2*; *Srebf1*, *sterol regulatory element binding transcription factor 1*; *Lxra*, *liver X receptor a*; *Rxra*, *retinoid X receptor a*; *Insig2*, *insulin induced gene 2*; *Zo1*, *zonula occludens 1*; *Ocln*, *occludin*; *Cldn*, *claudin*; *Akp*, *alkaline phosphatase*

Original blots presented in the manuscript.

Figure 3a. NR1D1

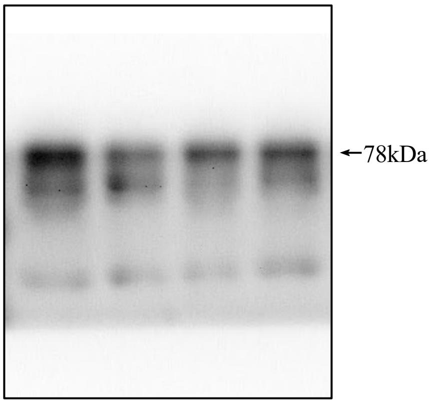

Figure 3a. NR1D2

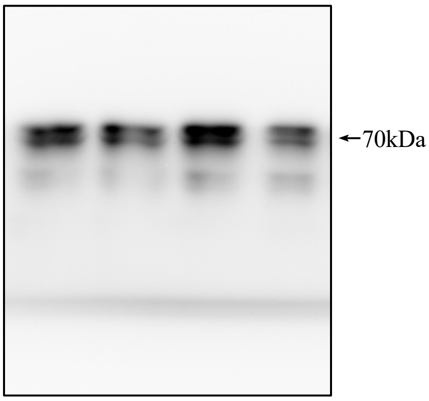

Figure 3a. INSIG2

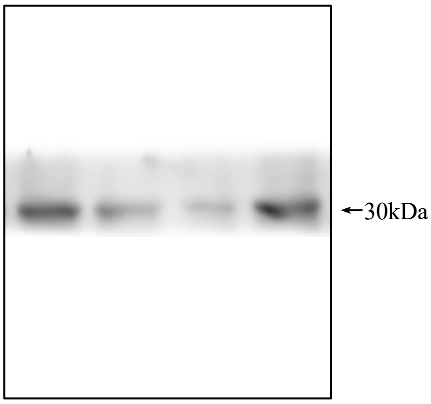

Figure 3a. SREBP1

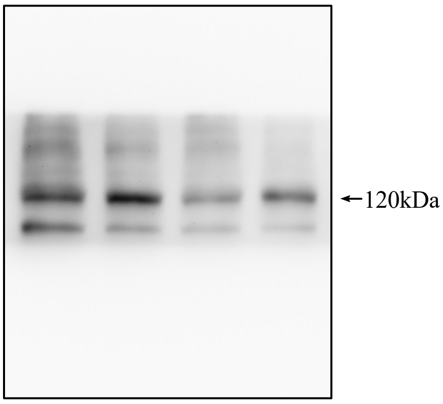

Figure 3a. LXR

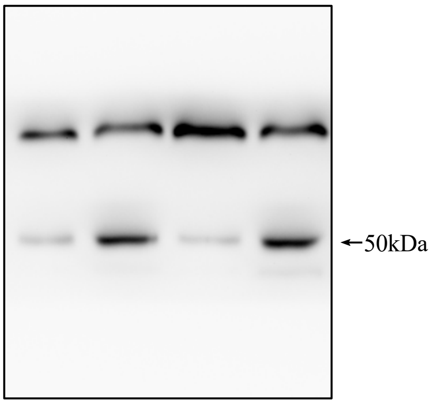

Figure 3a. RXR

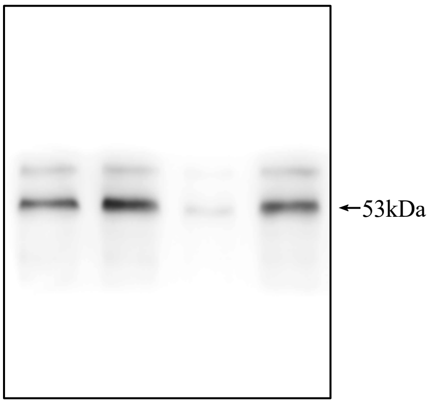

Figure 3a. GAPDH

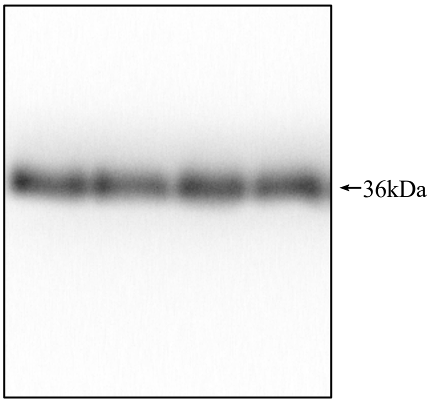

Figure 3c. NR1D1

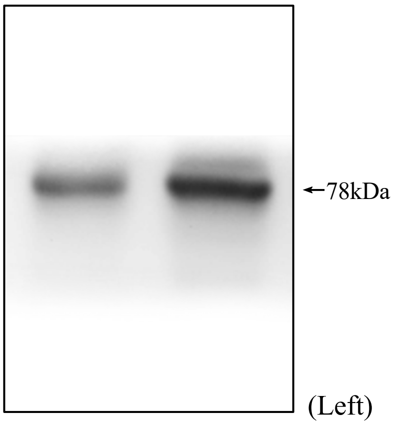

(Left)

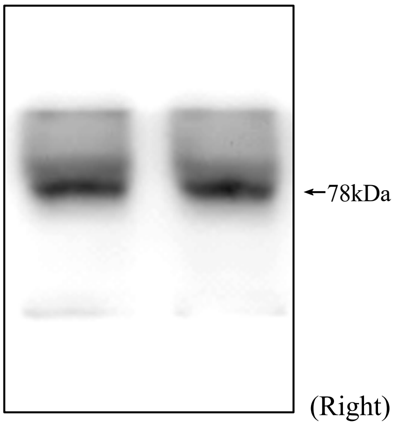

(Right)

Figure 3c. NR1D2

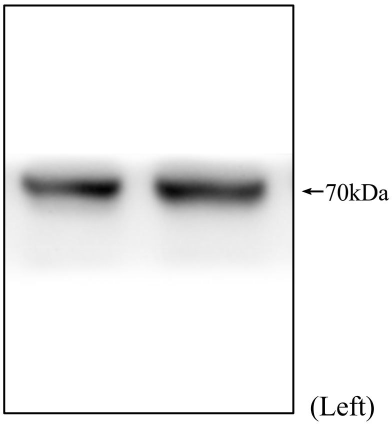

(Left)

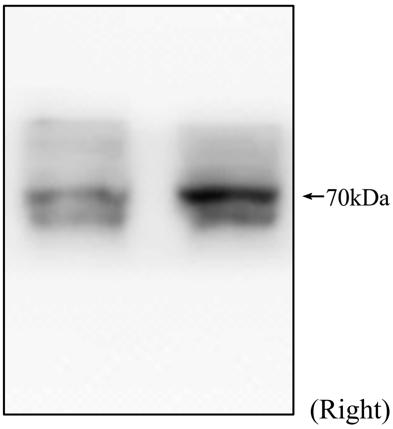

(Right)

Figure 3c. INSIG2

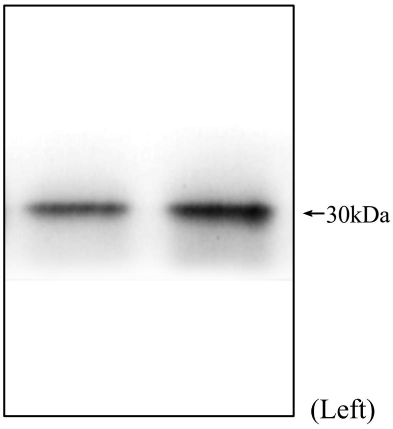

(Left)

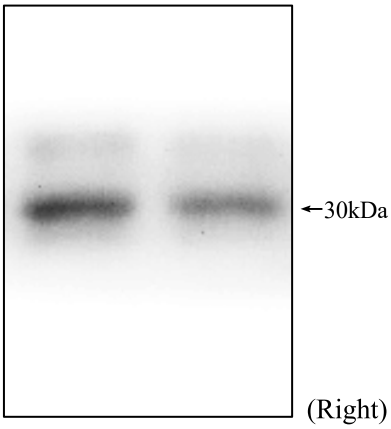

(Right)

Figure 3c. SREBP1

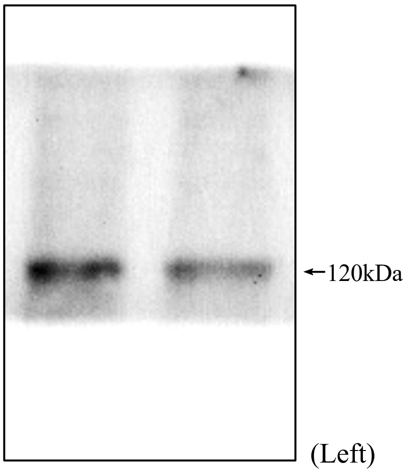

(Left)

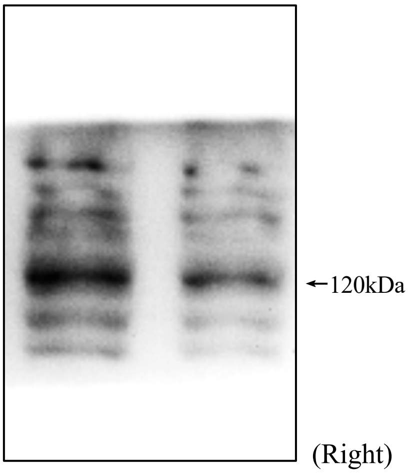

(Right)

Figure 3c. LXR

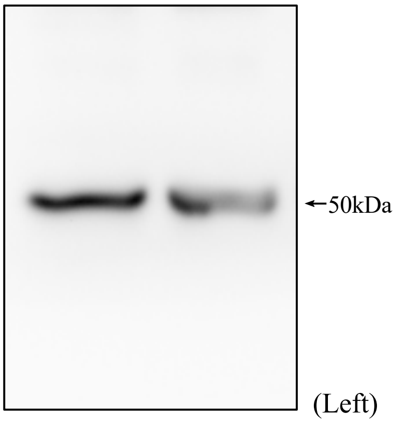

(Left)

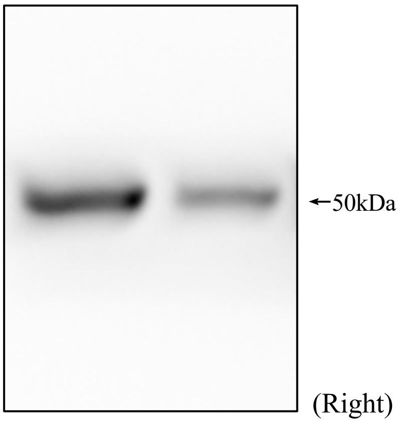

(Right)

Figure 3c. RXR

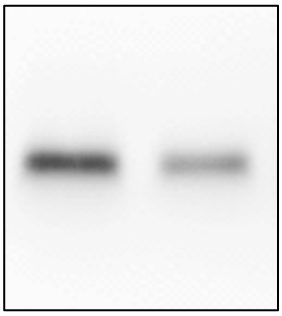

←53kDa

(Left)

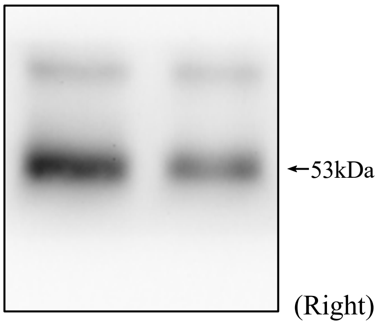

←53kDa

(Right)

Figure 3c. GAPDH

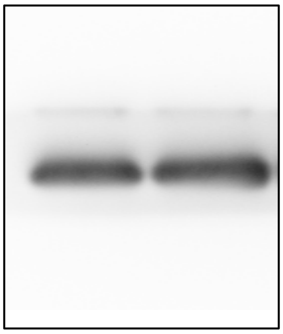

←36kDa

(Left)

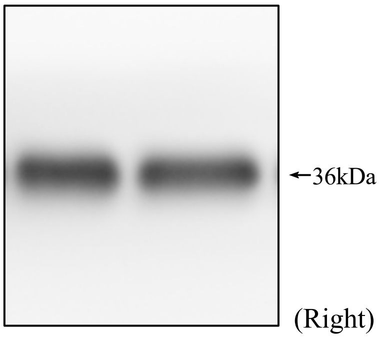

←36kDa

(Right)

Figure 3e. P-ERK

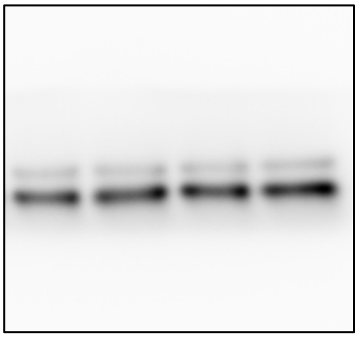

←44kDa
←42kDa

Figure 3e. ERK

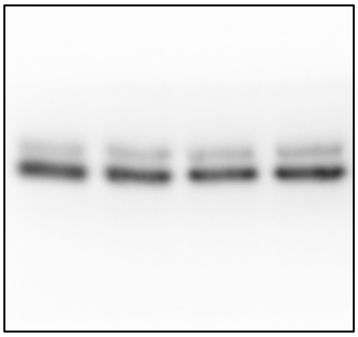

←44kDa
←42kDa

Figure 3e. P-AKT

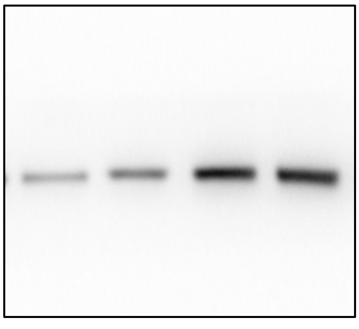

←60kDa

Figure 3e. AKT

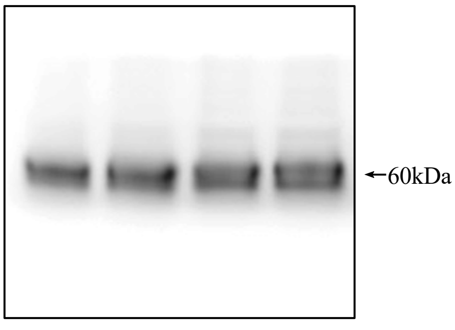

←60kDa

Figure 3e. NR1D1

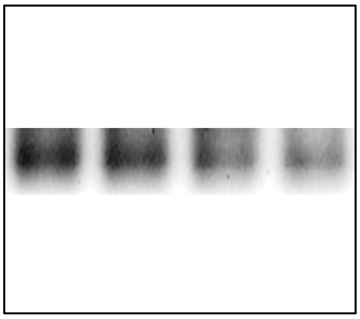

←78kDa

Figure 3e. NR1D2

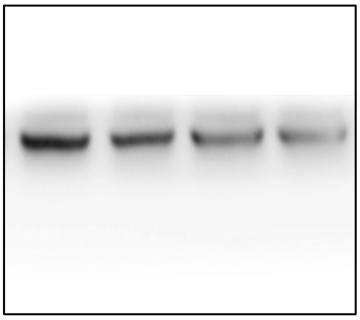

←70kDa

Figure 3e. INSIG2

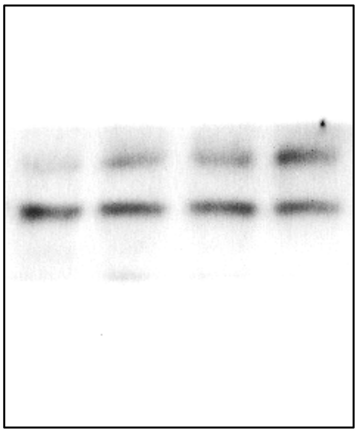

←30kDa

Figure 3e. SREBP1

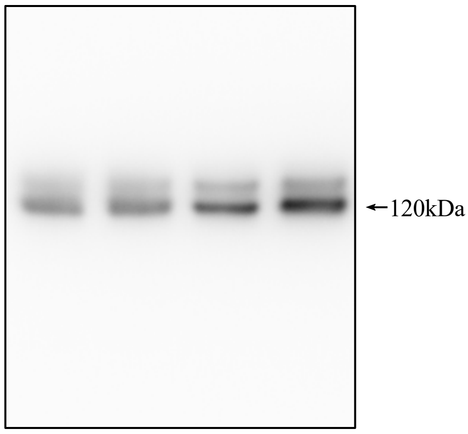

←120kDa

Figure 3e. LXR

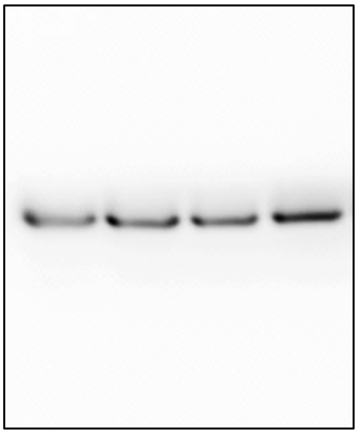

←50kDa

Figure 3e. RXR

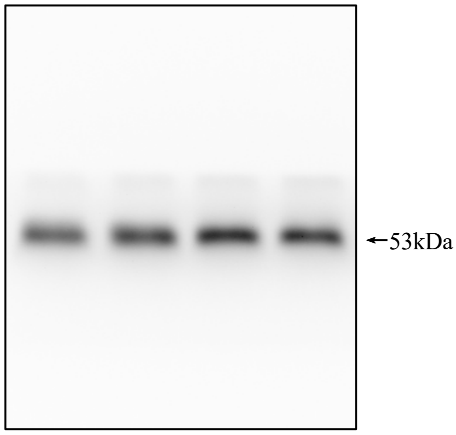

←53kDa

Figure 3e. GAPDH

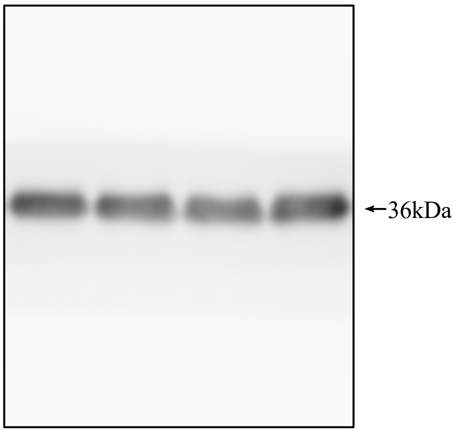

Figure 3f. GALR1

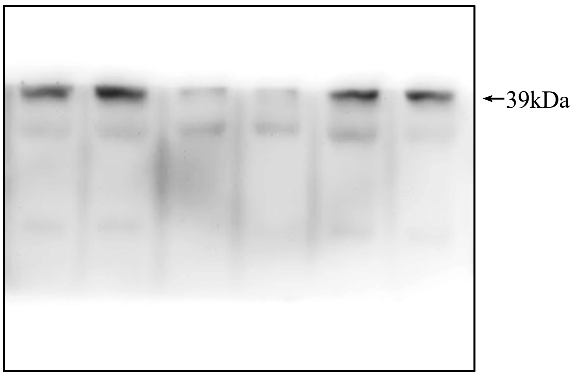

Figure 3f. GALR2

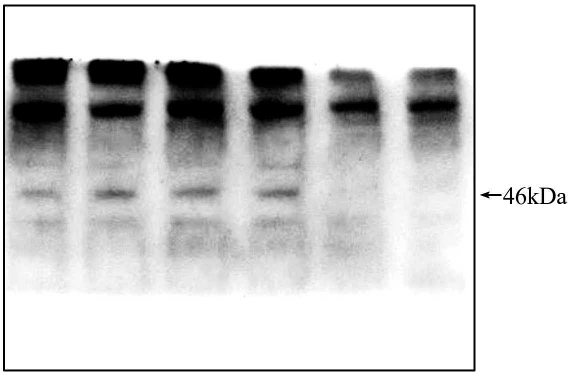

Figure 3f. P-ERK

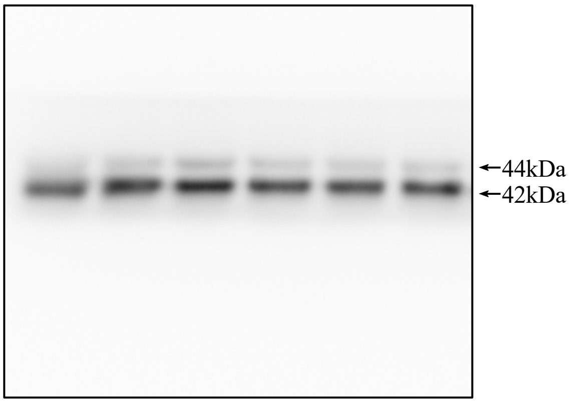

Figure 3f. ERK

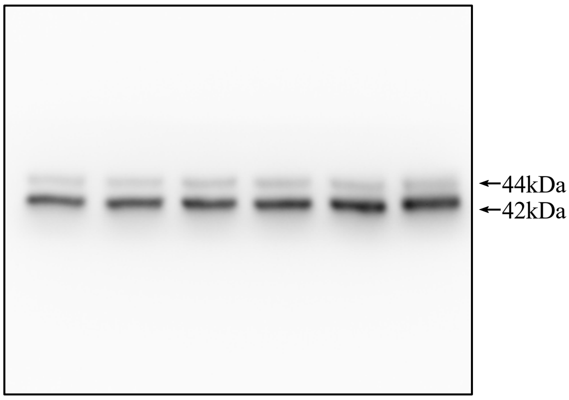

Figure 3f. P-AKT

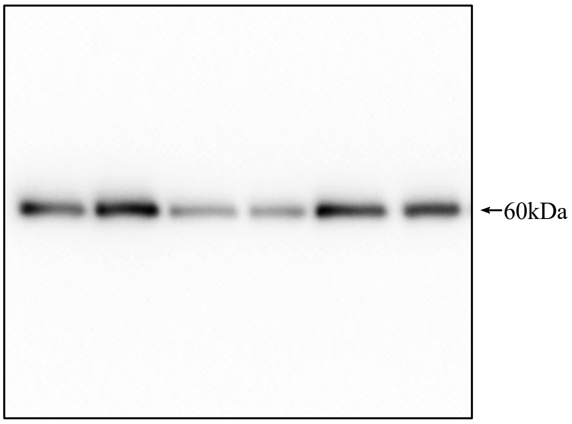

Figure 3f. AKT

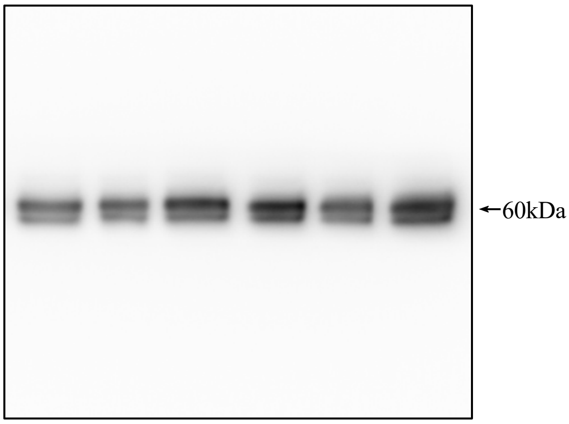

Figure 3f. NR1D1

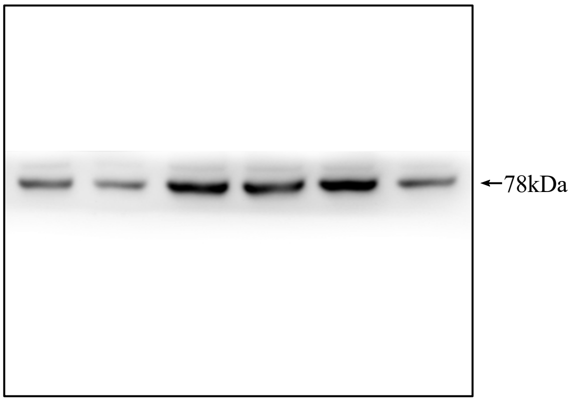

Figure 3f. NR1D2

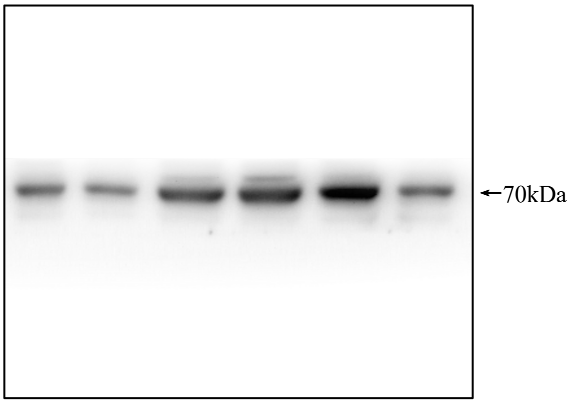

Figure 3f. INSIG2

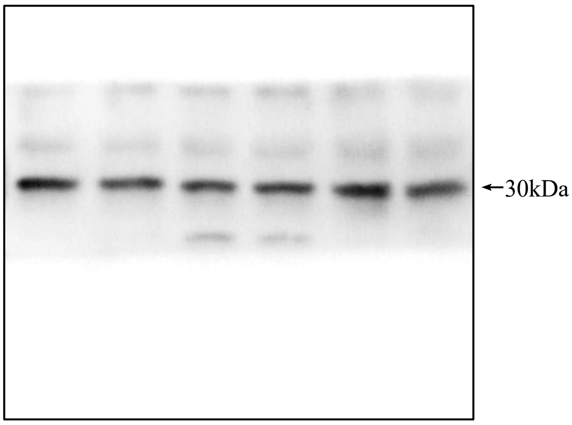

Figure 3f. SREBP1

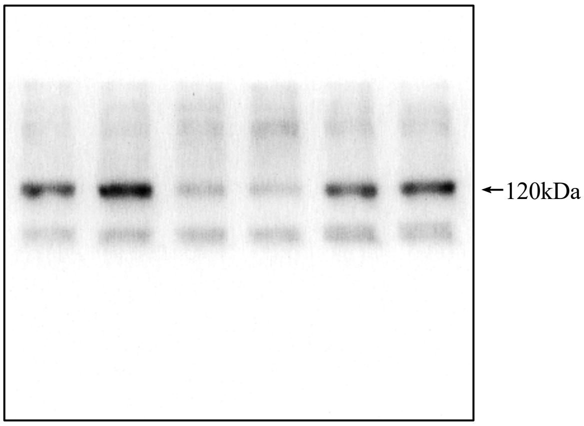

Figure 3f. LXR

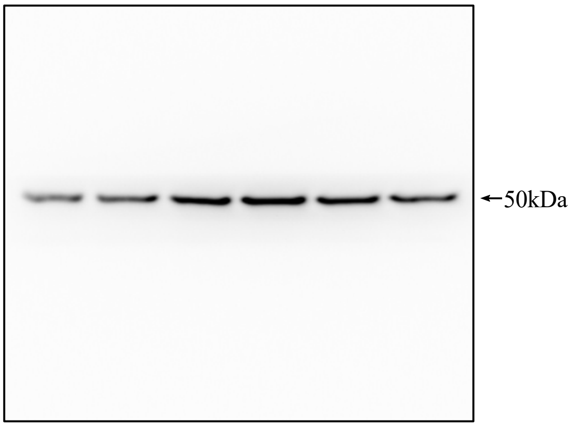

Figure 3f. RXR

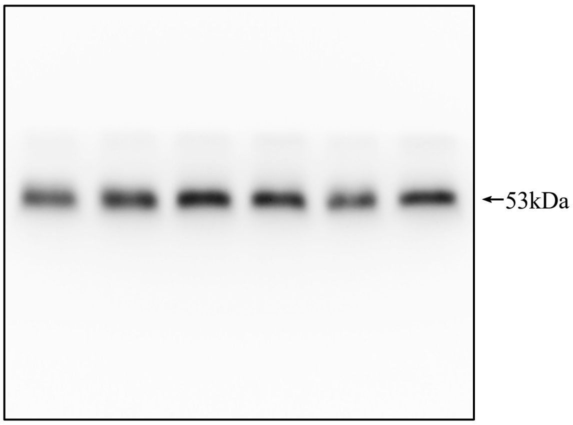

Figure 3f. GAPDH

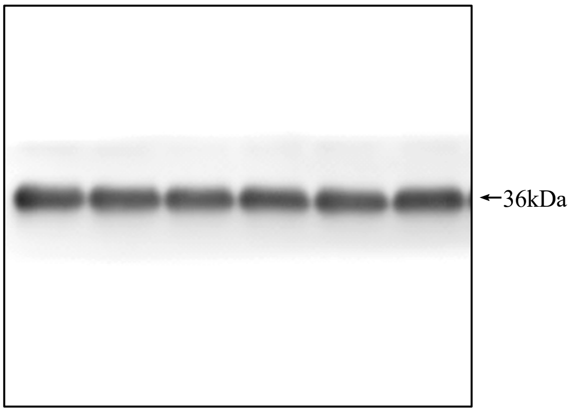

Figure 3g. GALR1

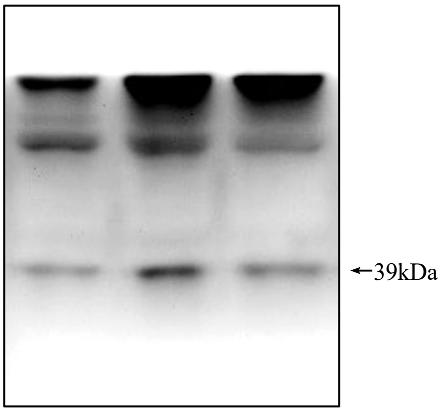

Figure 3g. GALR2

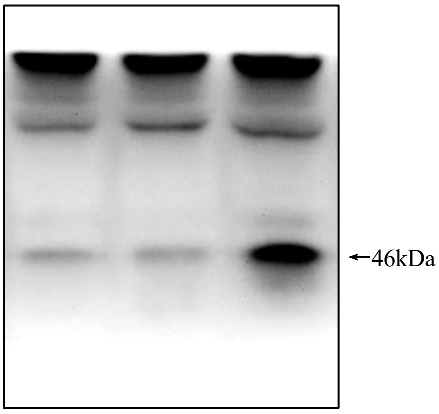

Figure 3g. P-ERK

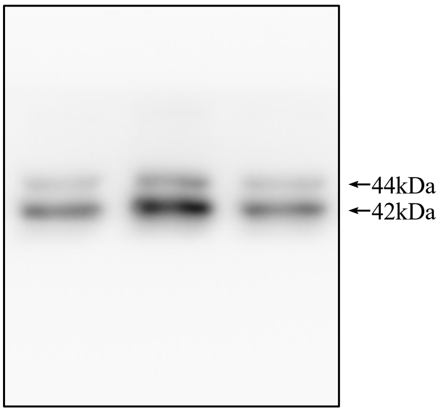

Figure 3g. ERK

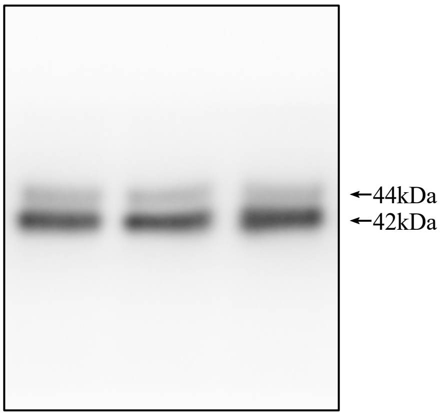

Figure 3g. P-AKT

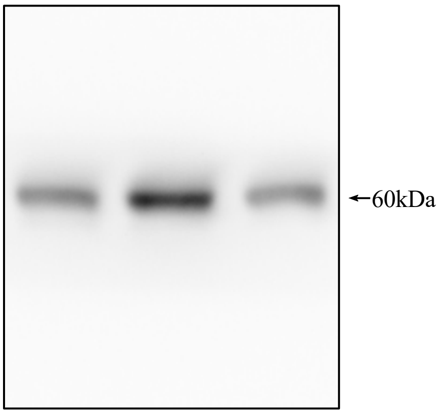

Figure 3g. AKT

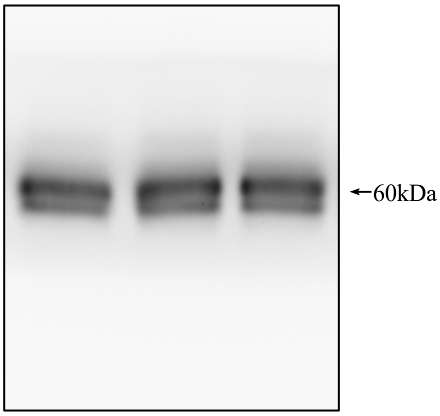

Figure 3g. NR1D1

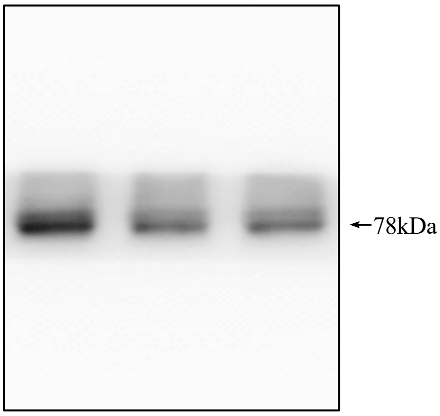

Figure 3g. NR1D2

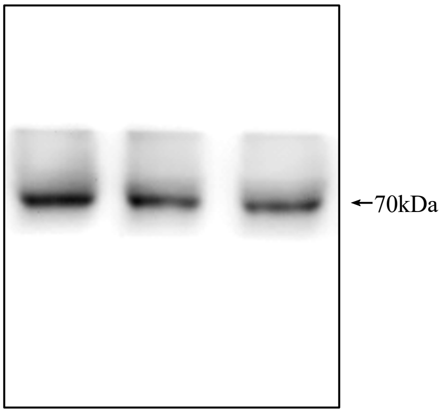

Figure 3g. INSIG2

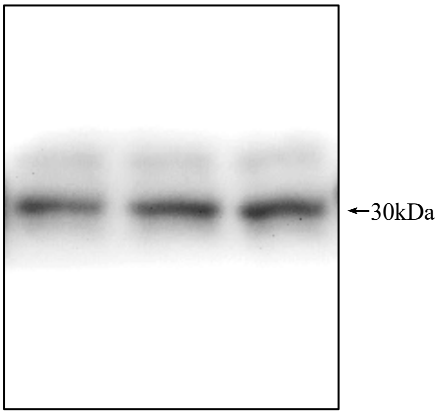

Figure 3g. SREBP1

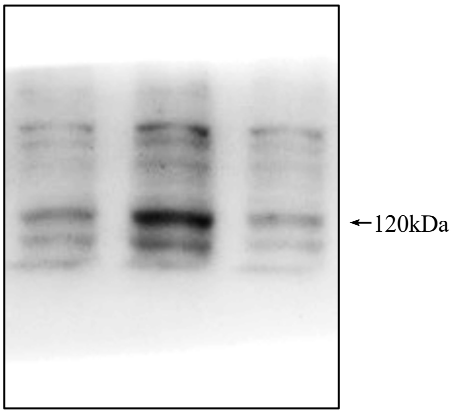

Figure 3g. LXR

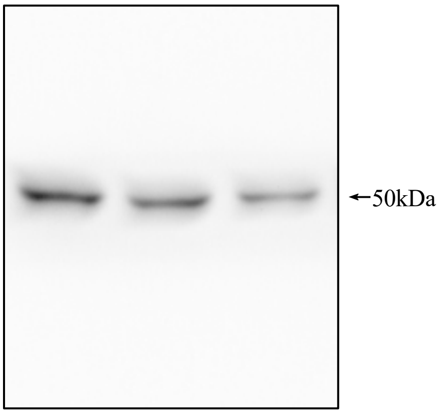

Figure 3g. RXR

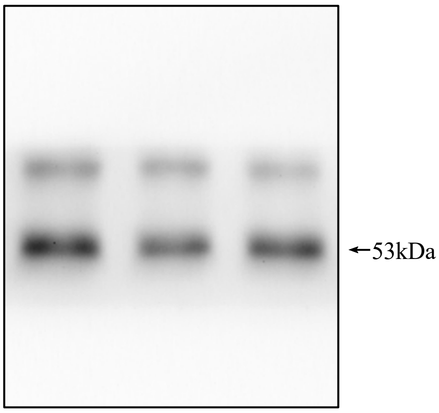

Figure 3g. GAPDH

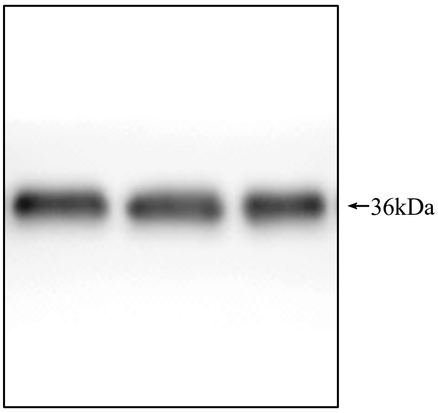

Figure 3h. P-ERK

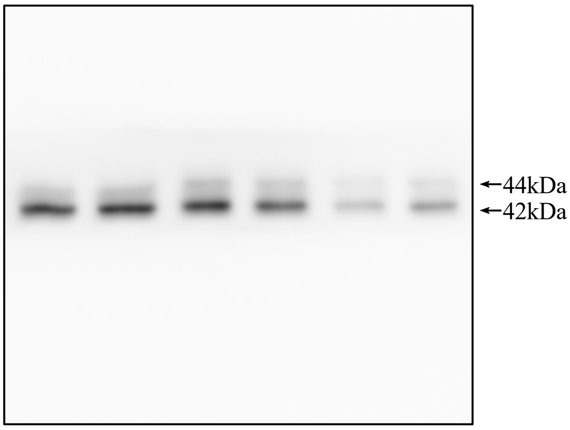

Figure 3h. ERK

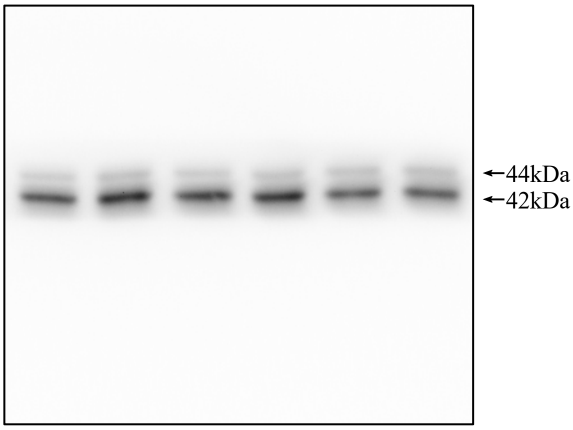

Figure 3h. P-AKT

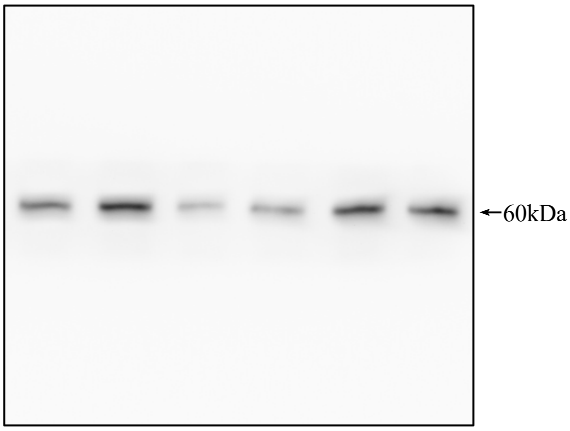

Figure 3h. AKT

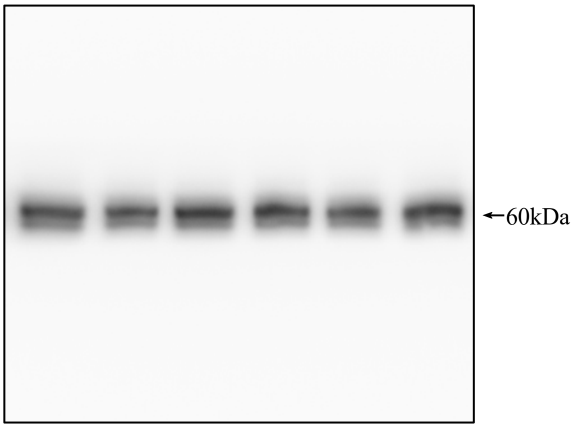

Figure 3h. NR1D1

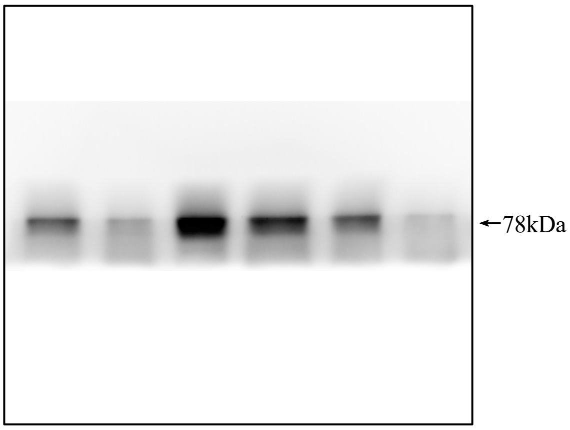

Figure 3h. NR1D2

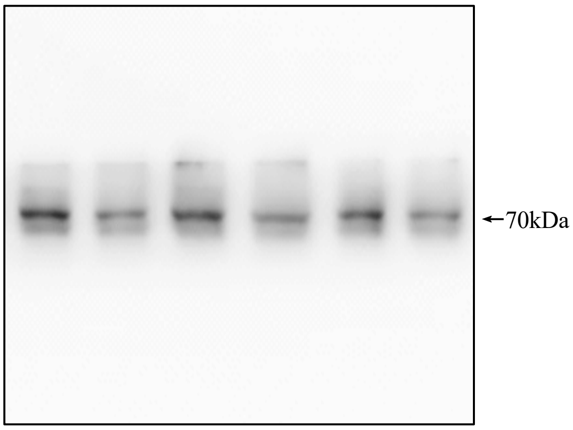

Figure 3h. INSIG2

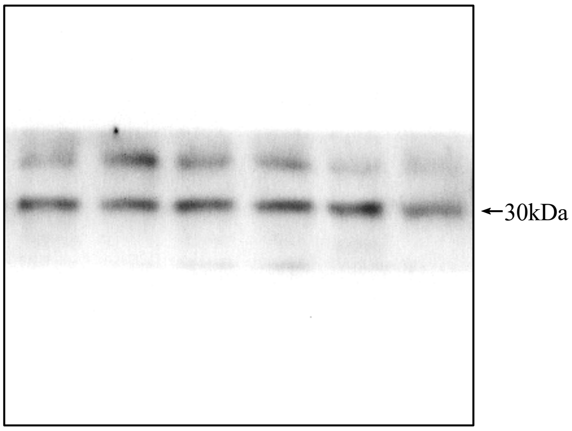

Figure 3h. SREBP1

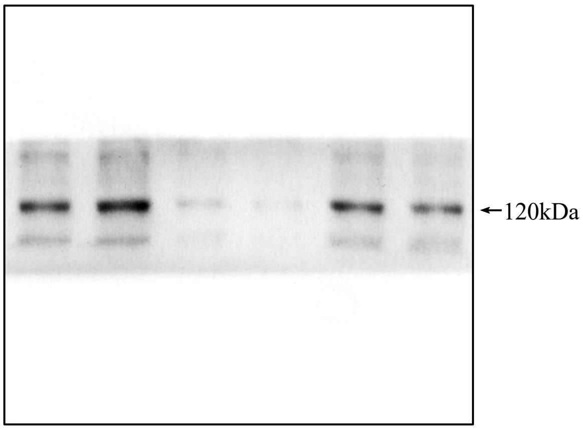

Figure 3h. LXR

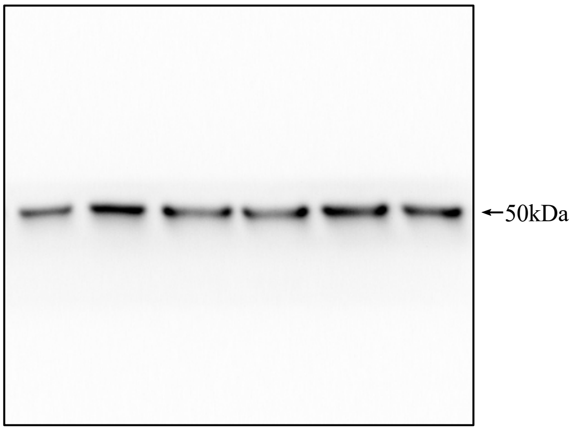

Figure 3h. RXR

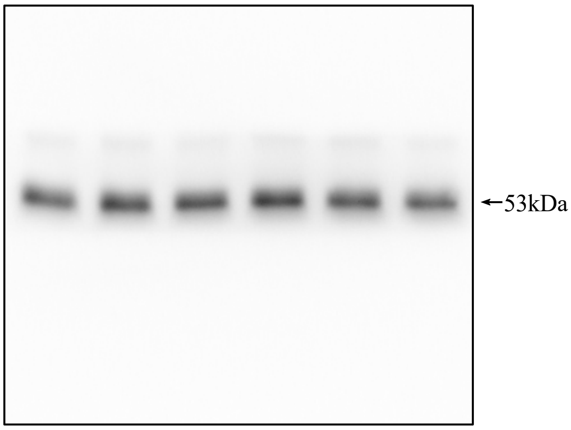

Figure 3h. GAPDH

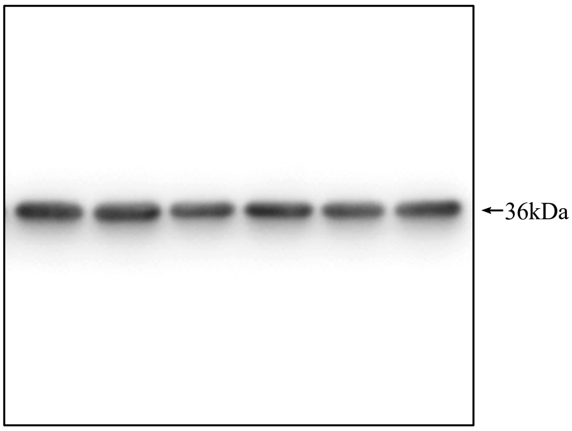

Figure 3i. GALR1

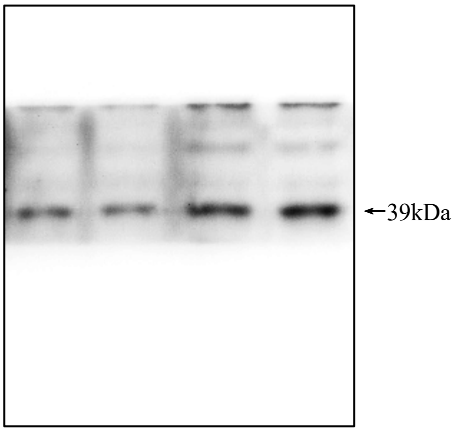

Figure 3i. P-AKT

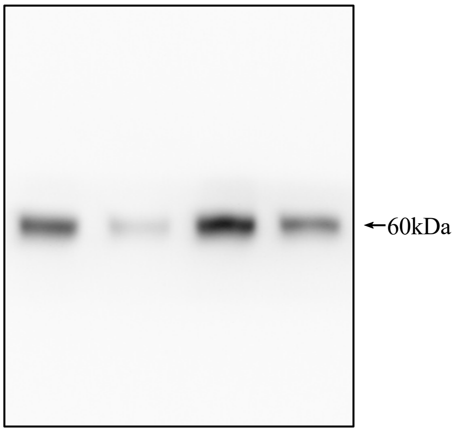

Figure 3i. AKT

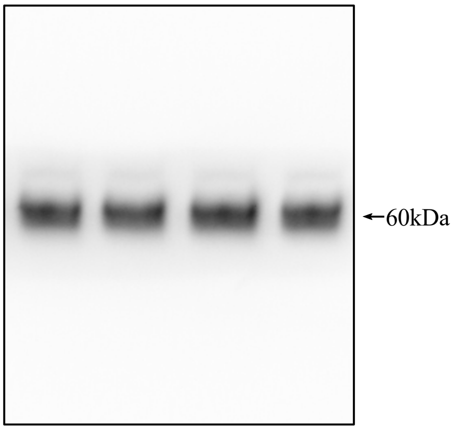

Figure 3i. NR1D1

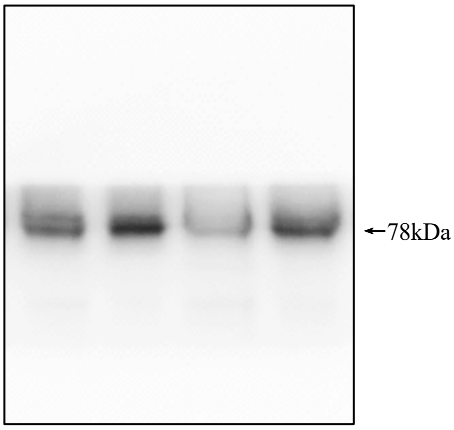

Figure 3i. NR1D2

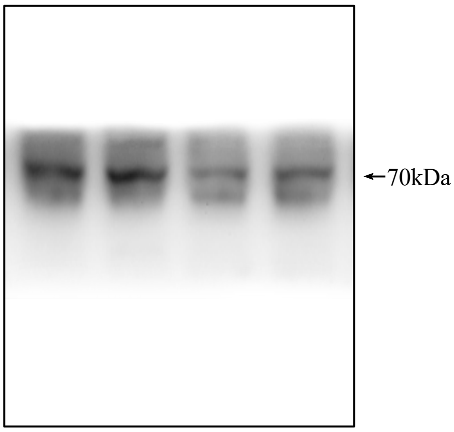

Figure 3i. INSIG2

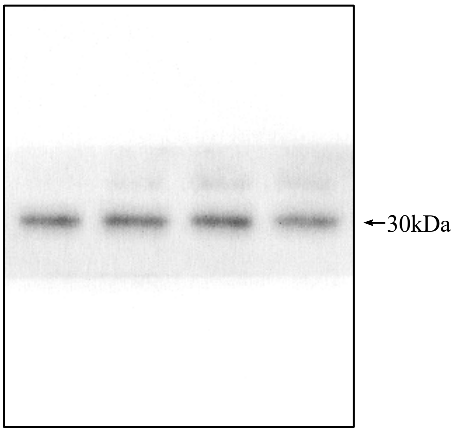

Figure 3i. SREBP1

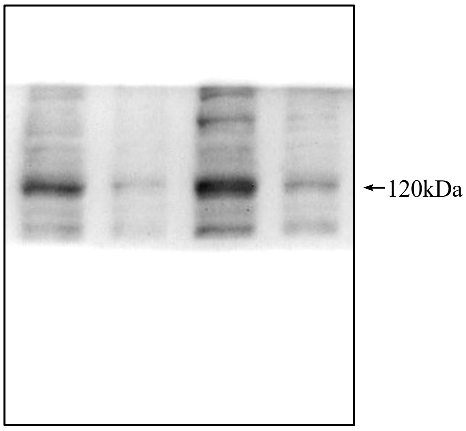

Figure 3i. LXR

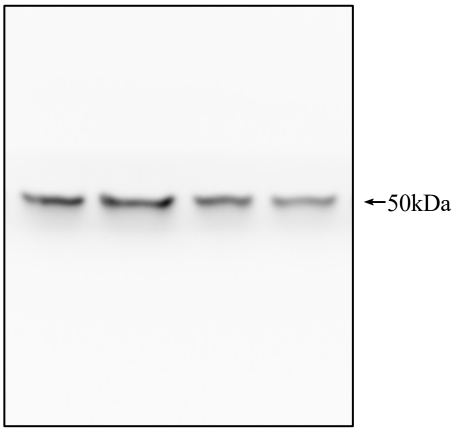

Figure 3i. RXR

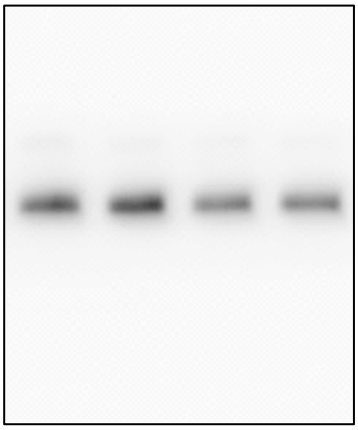

←53kDa

Figure 3i. GAPDH

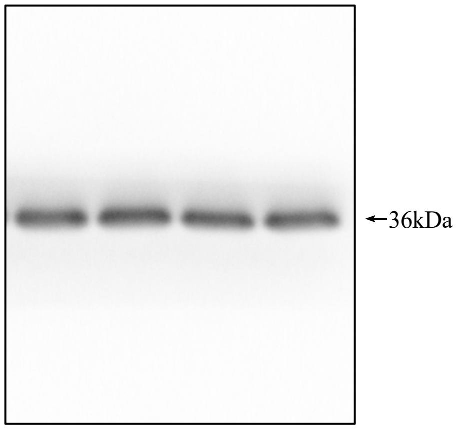

←36kDa

Figure 4f. NR1D1

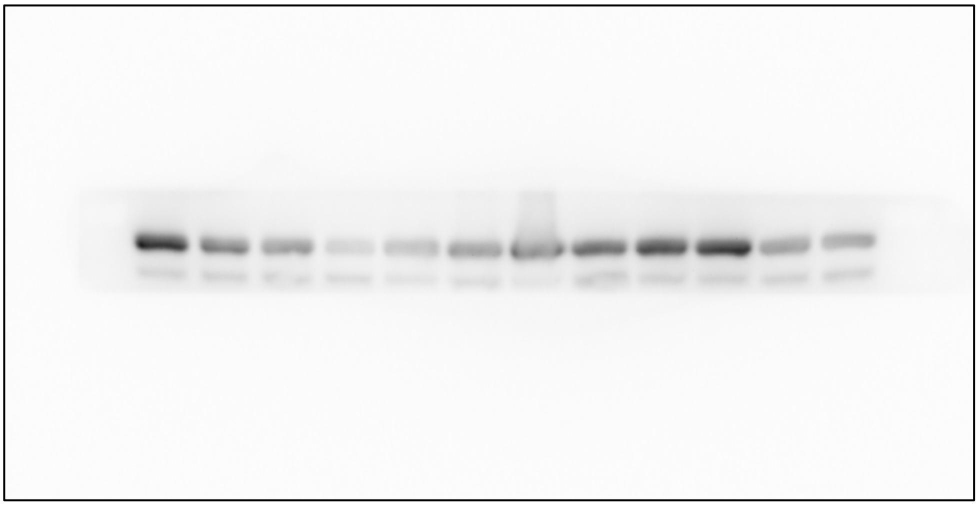

←78kDa

Figure 4f. SREBP1

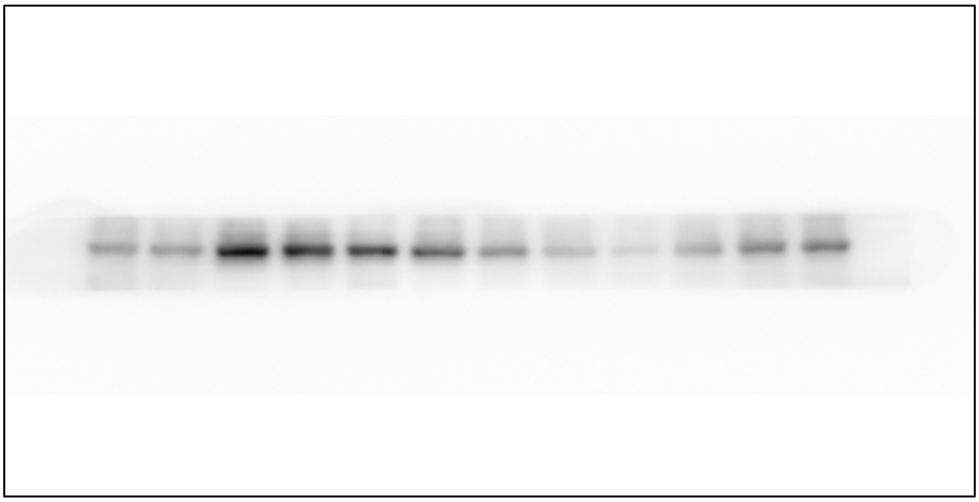

←120kDa

Figure 4f. GAPDH

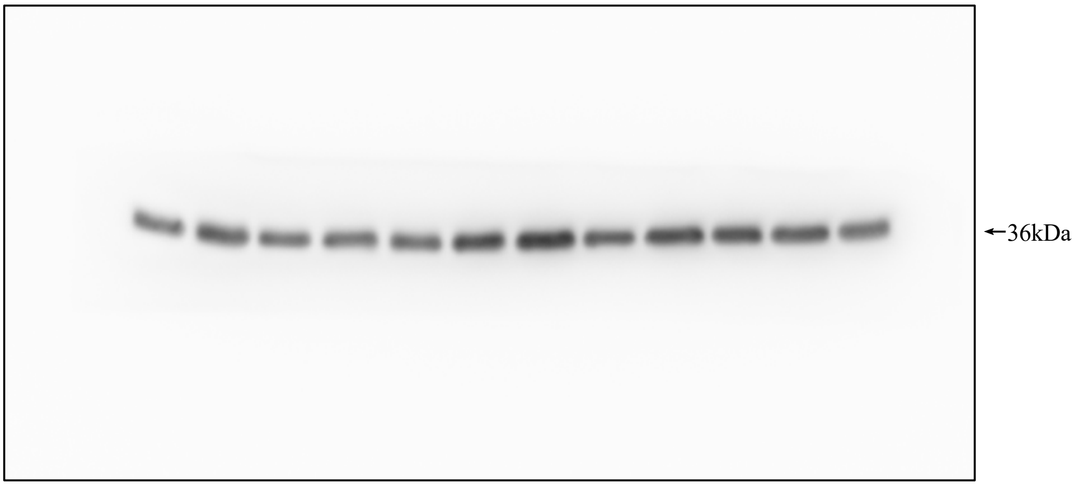

Figure 4i. NR1D1

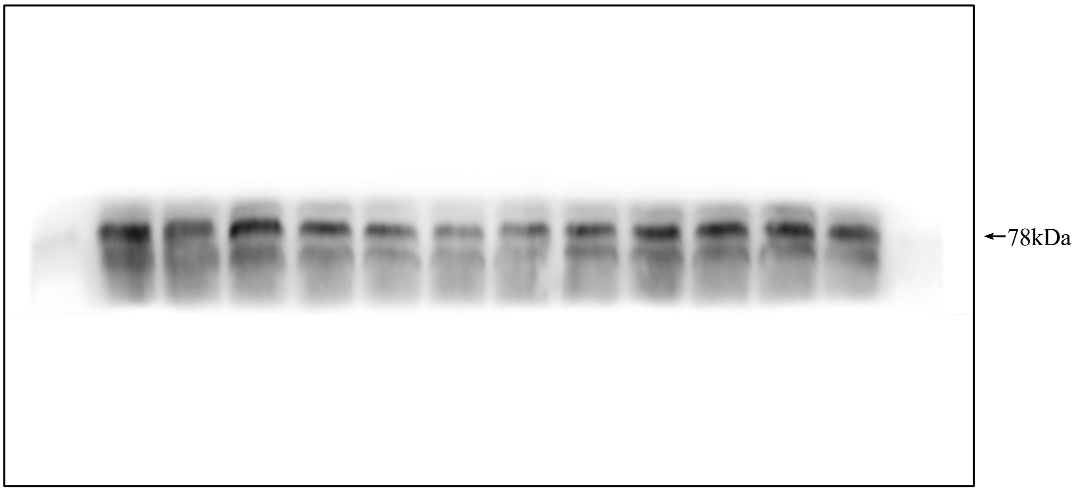

Figure 4i. SREBP1

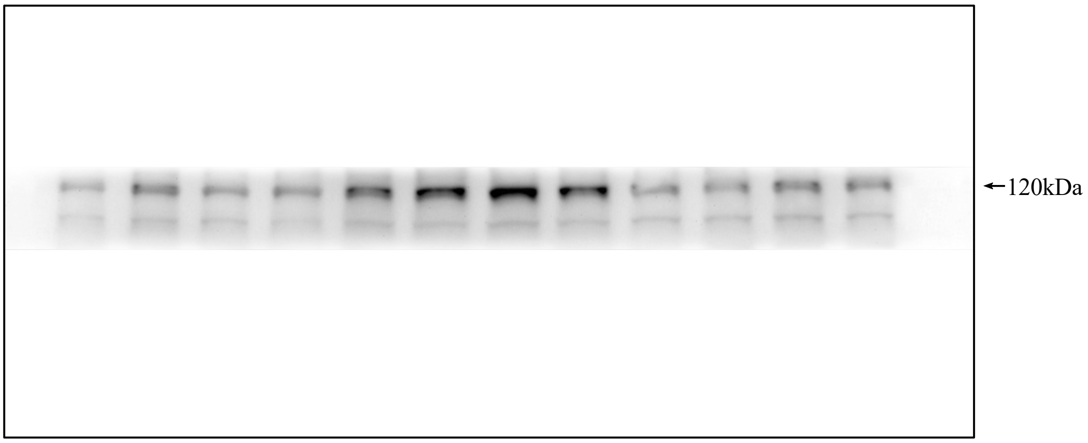

Figure 4i. GAPDH

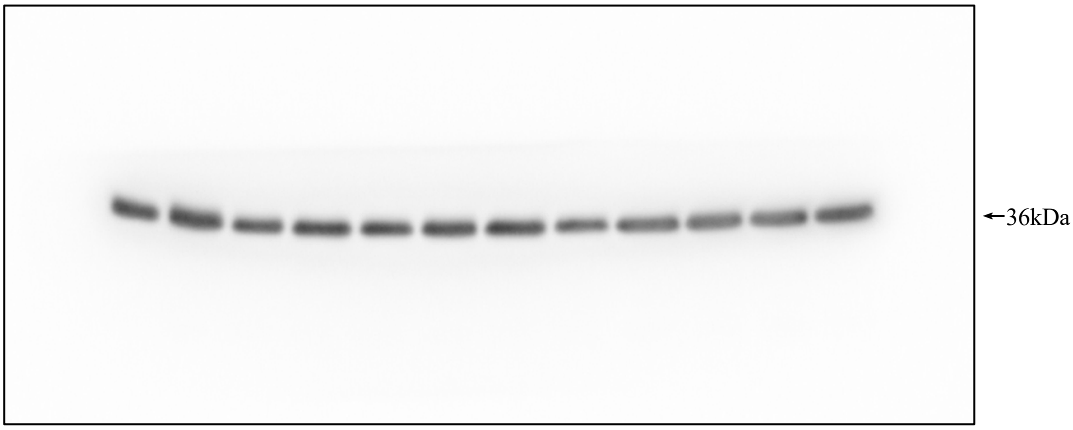

Supplementary Figure 1p. NR1D1

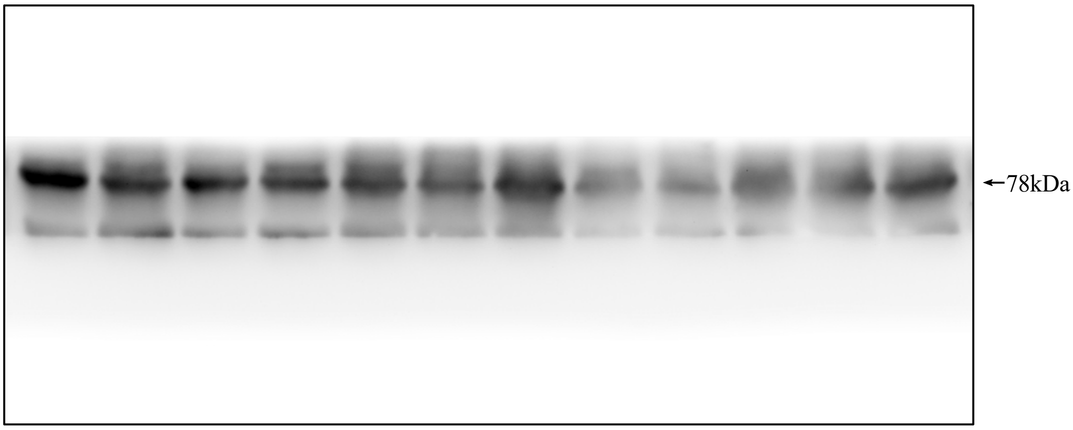

Supplementary Figure 1p. NR1D2

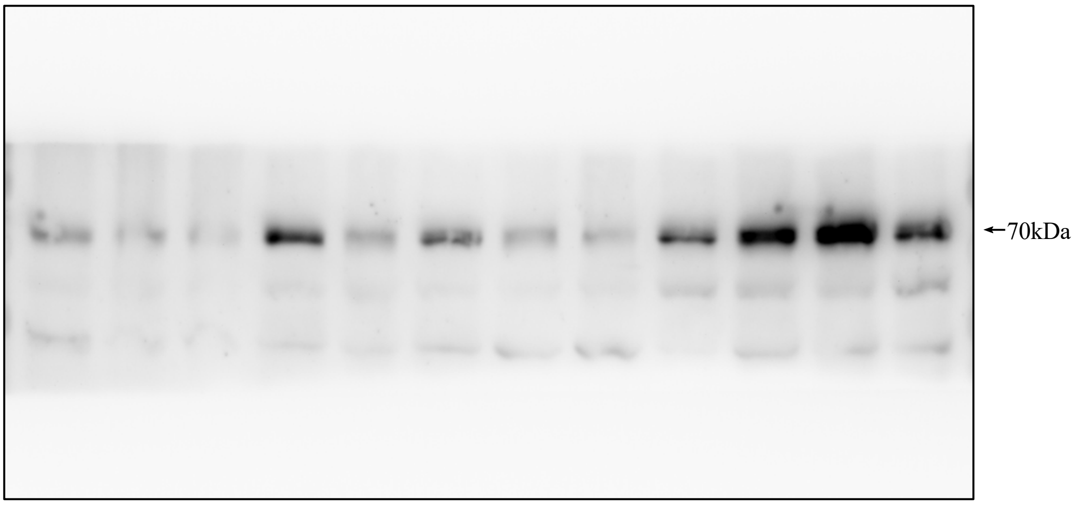

Supplementary Figure 1p. INSIG2

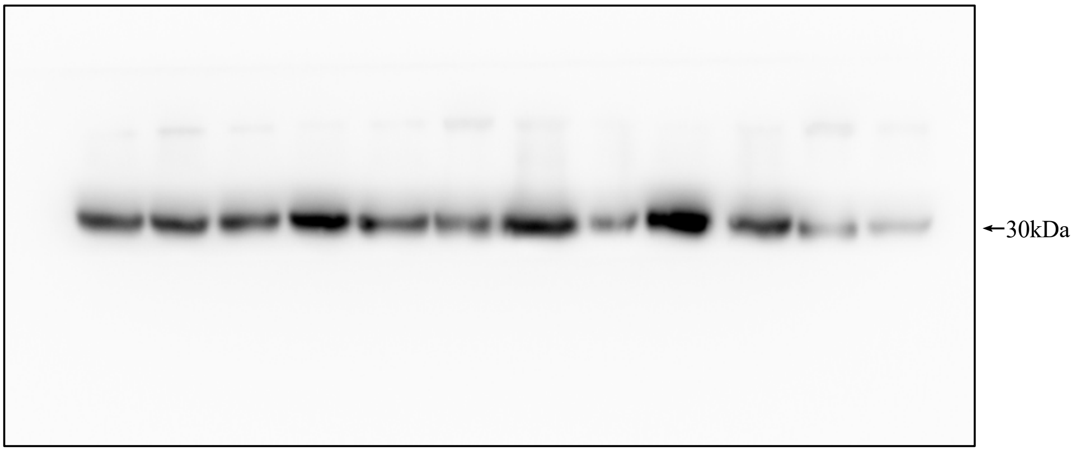

Supplementary Figure 1p. SREBP1

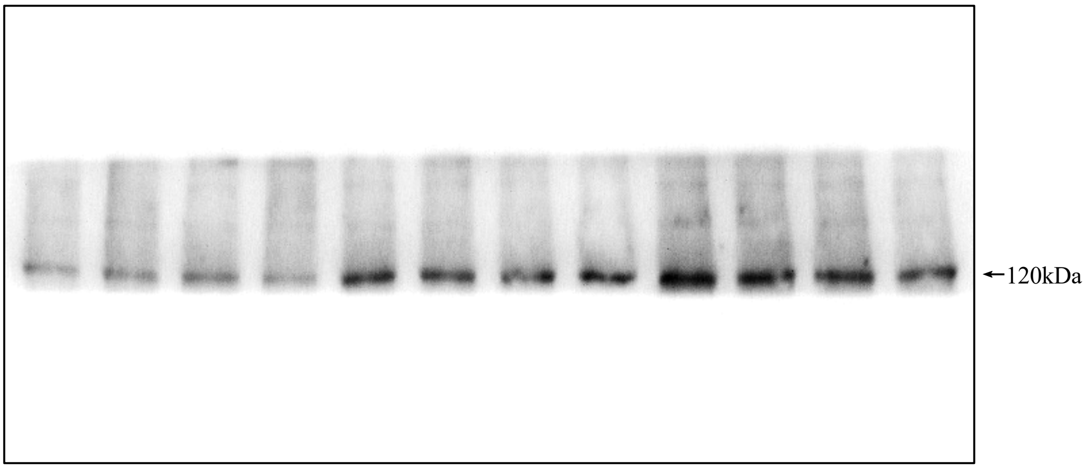

Supplementary Figure 1p. LXR

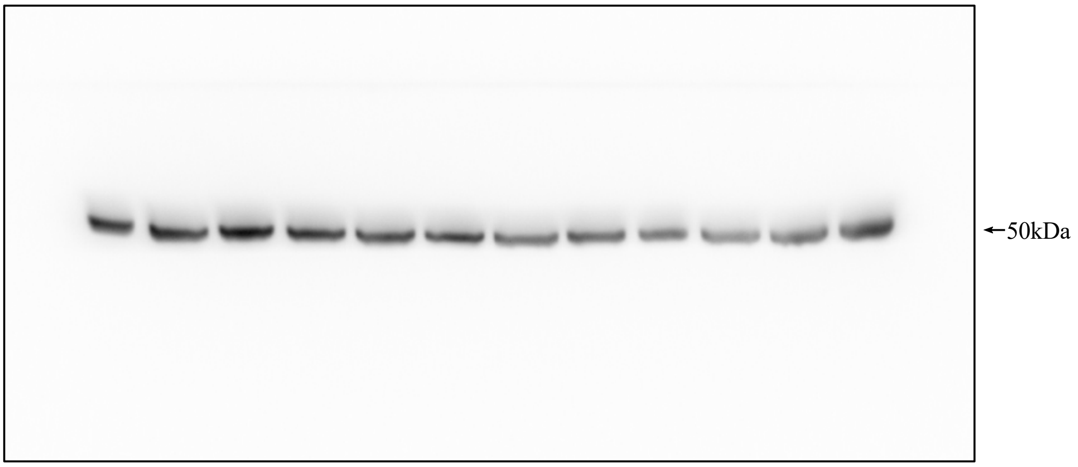

Supplementary Figure 1p. RXR

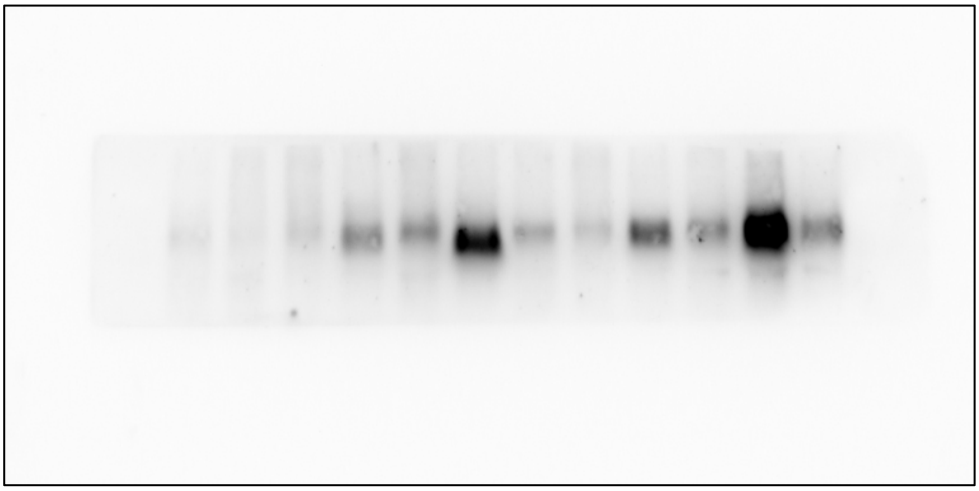

←53kDa

Supplementary Figure 1p. GAPDH

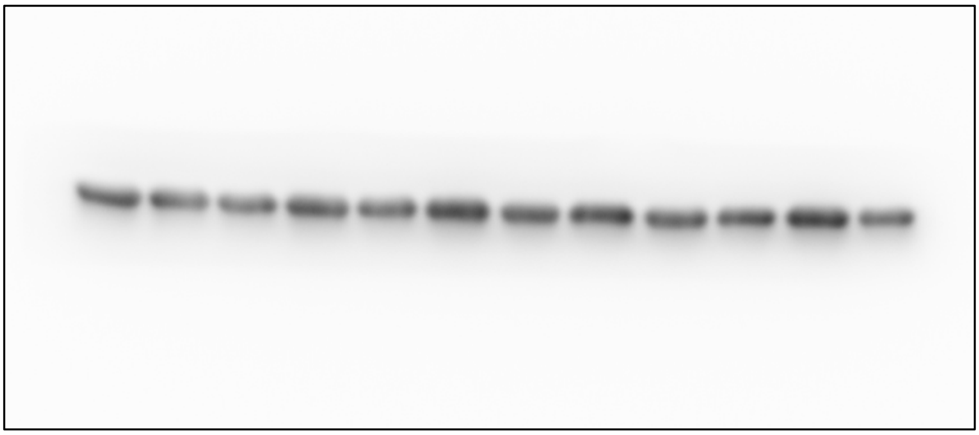

←36kDa
